# Effect of functional resistance training on the structure and function of the heart and liver in patients with non-alcoholic fatty liver

**DOI:** 10.1038/s41598-023-42687-w

**Published:** 2023-09-19

**Authors:** Ramin Jafarikhah, Arsalan Damirchi, Farhad Rahmani Nia, Seyyed Mohammad Taghi Razavi-Toosi, Afshin Shafaghi, Mostafa Asadian

**Affiliations:** 1 Department of Exercise Physiology, Faculty of Physical Education and Sport Sciences, University of Guilan, Rasht, Iran; 2 Medical Biotechnology Research Center, School of Paramedicine, Guilan University of Medical Sciences, Rasht, Iran; 3 Department of Cardiology, Heshmat Hospital, Cardiovascular Diseases Research Center, School of Medicine, Guilan University of Medical Sciences, Rasht, Iran; 4 GI Cancer Screening and Preventing Research Center (GCSPRC), Guilan University of Medical Sciences, Rasht, Iran; 5 Cardiovascular department, Razi Medical Education Center, Guilan University of Medical Sciences, Rasht, Iran

**Keywords:** Physiology, Health care, Medical research

## Abstract

The current study is of the quasi-experimental type, with a pre-and post-test design, and subjects were randomly assigned to one of two groups: control (n = 8) and experimental (test) (n = 8). Based on the patient's self-report and using daily diet control tables, the patient's diet planning percentage of energy supply was managed and controlled for 3 days. The protocol for functional resistance training for these circular exercises, including the squat, lunge, bear crawl, rock press, jumping jack, and back fly lunge, was performed three times per week without specialized apparatus. Ejection fraction (EF) and fractional shortening (FS) were measured before and after functional resistance training, using echocardiography. Liver Stiffness and steatosis were measured using FibroScan, and the liver function was determined using biochemical assays. The average age of patients in the control group and the test group were 46.02 ± 5.4 and 48.6 ± 2.51, respectively. Pre-test and post-test of the body mass index were 32.06 ± 5.06 and 30.02 ± 3.97, and for the body fat percentage were 33.65 ± 6.09 and 25.41 ± 4.99. In non-alcoholic fatty liver patients, due to functional resistance training, EF (p-value = 0.003) and FS (p-value = 0.03) significantly increased, and C-reactive protein (Hs-CRP) (p-value = 0.001), steatosis (p-value = 0.04), and stiffness (p-value = 0.01) decreased. According to the results and without considering clinical trials, functional resistance training affects the structure and function of the heart and Liver in NAFLD patients.

## Introduction

Non-alcoholic fatty liver disease (NAFLD) is one of the most prevalent liver diseases^1^. The global prevalence of NAFLD is 6 to 35%^2, 3^. NAFLD is less hazardous than nonalcoholic steatohepatitis (NASH) and does not typically progress to NASH or cirrhosis of the liver^4, 5^. Estimates indicate that between 30 and 40% of adults in the United States have NAFLD, and between 3 and 12% of adults have NASH^4^.

This disease is significant due to the destruction of liver cells, which can progress to cirrhosis, where liver transplantation is the only treatment^3^. Obesity, type 2 diabetes, overweight, and metabolic syndrome, are strong risk factors for NAFLD^6, 7^. Nonalcoholic fatty liver disease is a risky cause of liver cirrhosis, metabolic syndrome, cardiovascular disease (CV), and numerous other conditions^2^. Forty to fifty percent of patients with nonalcoholic fatty liver disease die from cardiovascular disease^2, 8, 9^. The association between NAFLD and CV complications such as coronary artery disease (CAD), atherosclerosis, and cardiac arrhythmias, as well as structural and functional alterations, has been demonstrated in prior research^10^. Patients with NAFLD frequently exhibit features of metabolic syndrome, which is associated with an increased cardiovascular risk^11^. Nearly 90% of NAFLD patients exhibit at least one symptom of metabolic syndrome, and 33% meet all diagnostic criteria for metabolic syndrome. Lack of physical activity is a recognized risk factor for fatty liver disease (FLD)^11, 12^.

Insulin resistance is a known cardiovascular risk factor. Physical activity reduces insulin resistance by enhancing insulin sensitivity in skeletal muscle and decreasing hepatic steatosis. Furthermore, physical activity increases energy expenditure. This can result in weight loss and a reduction in intrahepatic triglyceride levels. In addition, the benefits of physical activity have been demonstrated in patients with obese liver disease who are at risk for cardiovascular disease^10^.

Functional resistance training has been a part of the training program in some treatment methods for chronic heart and liver diseases. These exercises were previously used for therapeutic effects on metabolic syndrome and have had positive results in indicators of, Obesity, hyperglycemia, high blood pressure, and hypertriglyceridemia^13^. Also, the studies showed that functional resistance training increased parasympathetic excitability in the autonomic function of the heart and was reported as an anti-fibrillation effect in improving ventricular function^14^.

Functional training is gaining popularity in the fitness industry and is regarded as a superior alternative to conventional resistance training for enhancing various measures of muscular fitness, such as strength, endurance, coordination, and balance^12^. Since functional resistance training in inactive individuals and liver patients has been studied to a limited extent, and the feasibility of these exercises is acceptable and safe, this type of activity can be used as an alternative to conventional practice. In contrast to conventional training, this type of training has the potential to be more innovative in enhancing the performance of adults and applies to people of all ages and physical abilities^15^.

The cardiovascular system induced a significant adaptation to sports training. The most important of these adaptations include changes in the cardiovascular system's structural cellular and molecular signaling pathways that allow tissues to respond to the physical demands created by exercise^16^.

Consequently, this study aims to determine the effect of functional resistance training on the structure and function of the heart and liver in patients with non-alcoholic fatty liver disease.

## Methods

### Examine patients

Patients with NAFLD were randomly assigned to either a test (n = 8) or a control (n = 8) group. The demographic characteristics of participants are outlined in Table 1. The Participants were middle-aged men, 40 to 55 years old, suffering from non-alcoholic fatty liver disease, not suffering from cardiovascular and respiratory diseases, not smoking, not consuming more than 30 g of alcohol per night, not taking special medicinal supplements, and not adhering to a special diet, not having a history of diseases and orthopedic problems, not being prohibited from participating in sports activities and not having regular sports training in the previous year. This investigation was approved by the Ethics Committee of the Guilan University of Medical Science [IR.GUMS.REC.1399.454], and all participants provided written informed consent. All procedures were performed in accordance with the latest Declaration of the Guilan University of Medical Science. In this study, twenty men with non-alcoholic fatty liver, whose maladies were confirmed by a specialist physician and who volunteered to participate, were selected as a statistical sample of sixteen patients, who were randomly divided into two control and test groups, based on the research criteria.

**Table 1:** Demographic variables.

Demographic Variables		Control group^a^ (n = 8)	Test group (n = 8)	Levene’s test^b^	Independent t-test with Welch's corrections^c^	Independent t-test
Age (year)		46.20 ± 5.40	48.60 ± 2.51	0.026	0.394	
Body mass index	Pre-test	33.76 ± 1.02	32.06 ± 5.06	0.001	0.499	
	Post-test	33.16 ± 1.13	30.02 ± 3.97			
Body fat percentage %	Pre-test	34.57 ± 5.54	33.65 ± 6.09	0.971		0.809
	Post-test	31.17 ± 7.06	25.41 ± 4.99			

Data are presented as means ± SD.

^a^The control group did not receive any intervention, but the test group did functional resistance training group 24 sessions over 8 weeks three sessions per week at a local gym. Welch’s t-test was applied to compare two sets of data.

^b^Levene’s test is used to assess the equality of variances for two groups in pre-measurement.

^c^Welch's correction and independent t-tests were used in the case of inequality of variance and equality of variance, respectively.

### Echocardiography evaluation of the studied groups

Using echocardiography, the cardiac function was evaluated (Sonoscape Model P-15 device, China). Transthoracic echocardiography was used according to the standard method to evaluate cardiac function^17^. In both participant groups, identical echocardiograms were undertaken. Electrocardiography was performed concurrently with echocardiography to examine the heart's rhythm and rate. A specialist doctor who was unaware of the treatment protocol administered echocardiography examinations. Initially, two-dimensional images were captured from the perspective of the left ventricle's short axis in the mid-parasternal section. Then, M-mode images were acquired from the parasternal four long-axis levels of the papillary muscles of the left ventricle to determine the following parameters:

- Dimensions of the left ventricle after systole (LVESd, cm).
- Dimensions of the left ventricle after diastole (LVEDd, cm).

The left ventricle's ejection fraction (EF) and fractional shortening (FS) were calculated using the following formulas^18^.

$$\begin{gathered} {\text{EF }}\left( \% \right) \, = { 1}00 \, \times \, \left[ {\left( {{\text{LVD}}_{{\text{d}}}^{{3}} - {\text{LVS}}_{{\text{d}}} } \right)^{{3}} /{\text{LVD}}_{{\text{d}}}^{{3}} } \right] \hfill \\ {\text{FS }}\left( \% \right) \, = { 1}00 \, \times \, \left[ {\left( {{\text{LVD}}_{{\text{d}}} - {\text{LVS}}_{{\text{d}}} } \right)/{\text{LVD}}_{{\text{d}}} } \right] \hfill \\ {\text{Left ventricular end systolic volume }}\left( {{\text{LVV}}_{{\text{s}}} \& {\text{ mL}}} \right) \, = { 1}.0{47 } \times \, \left( {{\text{LVS}}_{{\text{d}}} } \right)^{{3}} \hfill \\ {\text{Left ventricular end diastolic volume }}\left( {{\text{LVV}}_{{\text{d}}} \& {\text{ mL}}} \right) \, = { 1}.0{47 } \times \, \left( {{\text{LVD}}_{{\text{d}}} } \right)^{{3}} \hfill \\ {\text{Strokevolume }}\left( {{\text{SV }}\& {\text{ mL}}} \right) \, = {\text{ Diastolic volume }}{-}{\text{ Systolic volume}} \hfill \\ {\text{Cardiac outputs }}\left( {{\text{CO }}\& {\text{ mL}}/{\text{min}}} \right) \, = {\text{ EF }} \times {\text{ Heart rate}} \hfill \\ \end{gathered}$$

### Assessment of steatosis and stiffness in the examined categories

In clinical practice, transient elastography has evolved into a precise method and noninvasive instrument for evaluating hepatic steatosis and in terms of hepatic fibrosis. In this investigation, transient elastography was utilized by previously accepted procedures^1^. Liver steatosis and stiffness were assessed by (Fibro Touch- FT 100) subjectively, steatosis can be categorized as minimal, moderate, or severe. A minor modification was made to the assessment and grading of liver stiffness and steatosis based on previous studies. This study defined NAFLD as controlled attenuation parameter (CAP) scores of 240 dB/m or higher (S1, sensitivity fixed cut-off at 90%) mild, 265 moderate, and 295 severe. For sensitivity analysis, NAFLD was also defined as CAP scores of 295 dB/m or higher (optimizing cut-off sensitivity and specificity). The values for liver stiffness were 7.3 a, F0–F1, 9.7 F2, 12.4 F2–F3, 17.5 F3–F4, and 17.5-F4^1^.

### Blood variables in the studied groups

Serum levels of glucose, triglyceride, cholesterol, high-density lipoprotein cholesterol (HDL-C): low-density lipoprotein cholesterol (LDL-C) ratio, high-sensitivity C-reactive protein (HS-CRP), alanine transaminase (ALT), and aspartate aminotransferase (AST) were measured in outsourcing to an approved pathology laboratory by the University of Medical Sciences. The enclosed body fat percentage is measured and calculated by measuring the thickness of the skinfold in three areas of the thigh, chest, and abdomen using a (Lafayette Scientific Industries skin-fold calipers Model 01127; USA). All measurements were taken from the right side of the subject's bodies.

### Cardiac rhythm monitoring for arrhythmia detection

Holter monitoring of the heart rhythm was performed to detect cardiac rhythm within 24 h^19^. The Holter (American Mortara model H3+ device) was used to detect heart rate (HR), atrial fibrillation (AF), premature ventricular contraction (PVC), and premature atrial contraction (PAC). Electrodes on the thorax receive heart activity signals and transmit them to the device. During the cardiac monitoring, patients were instructed to refrain from bathing and swimming. These signals were captured by the device and transmitted to the operator in analog form. The operation of the device was thoroughly explained to the patient.

### Training protocol

The training protocol was implemented based on the previous study with a small modification^11^. Volunteers in both the control and test (intervention) groups submitted a consent form^20^, and all the test group volunteers were trained in the training protocol. The test group participated in twenty-four sessions over 8 weeks (3 sessions per week on nonconsecutive days) at a local facility during the winter of 2021.

**Figure 1:**
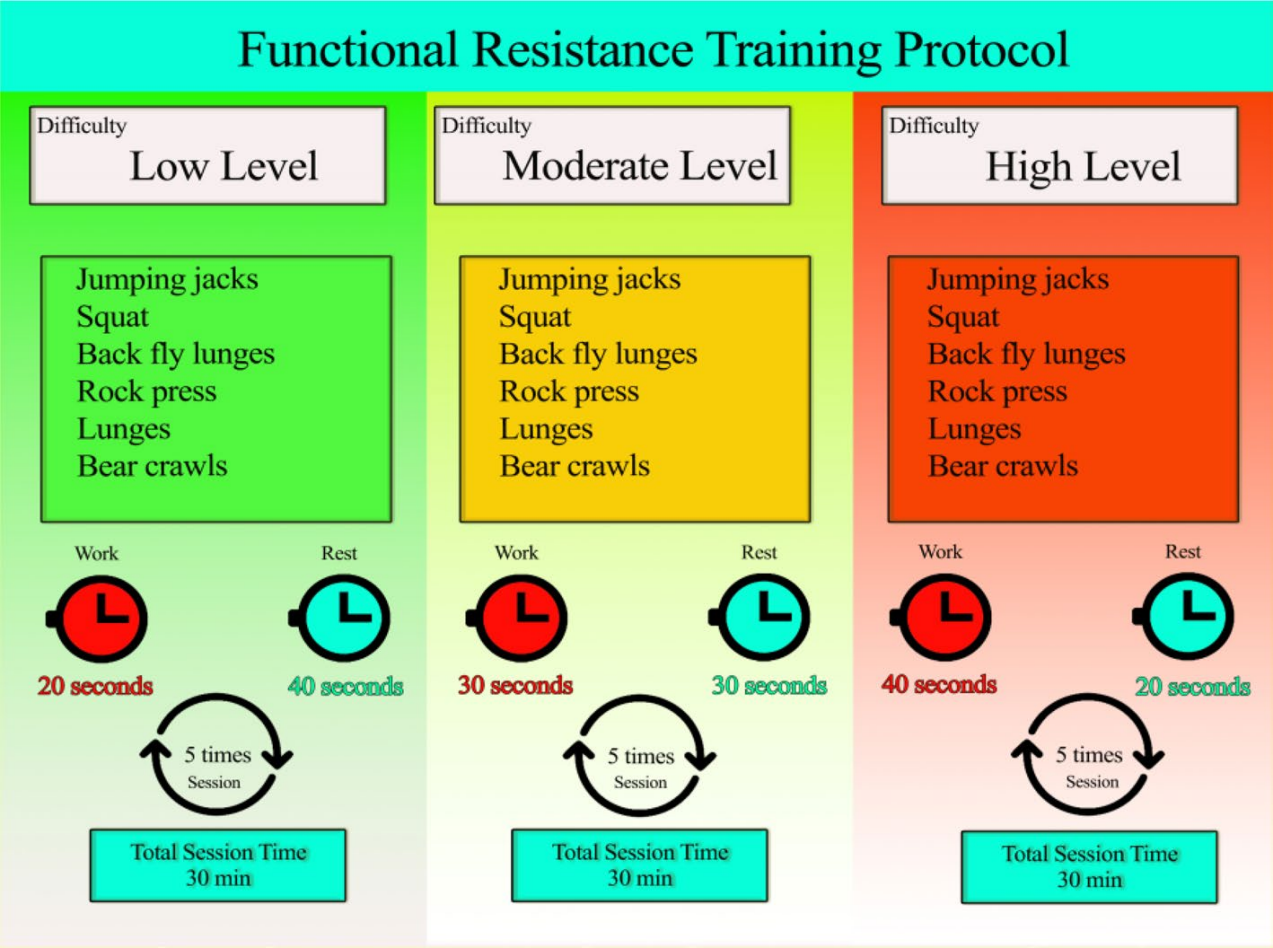
Functional resistance training.

These circular exercises include squats, lunges, bear crawls, rock presses, jumping jacks, and back fly lunges, without the need for any specialized apparatus. The training was in the form of bodyweight exercises and the program of the training session included three levels of load (low, moderate, and high). Low-level exercises were performed for two weeks (3 sessions per week), and each session consisted of five exercises (squat, lunge, bear crawl, rock press, jumping jacks, and lunge back fly) that consisted of 20 s of work and 40 s of rest. Moderate-level and final three high-level exercises were also held with three sessions per week, which were performed according to the low-level exercises, but for the moderate-level, we had 30 s of work and 30 s of rest and for 40 s of work, and 20 s of rest for the high-level exercises (Fig. 1). Finally, the training session ends with a general body cool-down^21^.

All steps of measuring load and training time were done under the supervision of a sports specialist (researcher). The load of physical activity in the test group was chosen from 12 to 14 based on the Borg scale (20–6) rating of perceived exertion (RPE).

### Statistical analysis

All data were presented as mean ± SD. The statistical analyses were accomplished with Statistical Package for the Social Sciences (SPSS) (version 25.0 software; SPSS, Chicago, IL); and graphs were drawn with GraphPad Prism version 9 software. To confirm the normal distribution of data, the Shapiro–Wilk test was accomplished (p-value > 0.05 was considered as the normal distribution). The one-way repeated measures analysis of variance (ANOVA) was utilized to examine the trend of variables over time between the two groups. Then to compare the two test and control groups at separate times, the independent t-test and Mann–Whitney test were used if the variable was normal and non-normal, respectively. If variables were normal, comparing pre- and post-measurements was done by the dependent t-test, and the Wilcoxon test was used, otherwise. Also, a p-value < 0.05 was considered statistically significant^22^.

## Results

### Statistical indicators

The average participant's age (years) was 47.40 ± 4.17, their height (m) was 1.79 ± 0.06, their weight (kg) was 106.80 ± 13.08, their body mass index was 32.91 $\pm$ 3.55, and their body fat percentage was 34.11 ± 5.51. The average age (years), body mass index (BMI), and body fat percentage at the outset of the study did not differ significantly between the control and test groups. The pre-test and post-test BMI differences between the control and test groups were not statistically significant. However, the difference in average body mass index between the pre-test and post-test was significant (p-value < 0.05) in both the control and test groups. The average percentage of body fat did not differ significantly between the control and test groups on either the pre- or post-test. Also, the difference between the average percentage of body fat in the pre-test and post-test in the control group was not significant, whereas this difference was significant in the test group, i.e., the average percentage of body fat in the test group differed significantly between the pre-test and post-test.

### Functional resistance training affects the trends of change in echocardiographic parameters

Based on the results of the repeated measures ANOVA, the significance in time means that the change of time from the pre- to the post-test caused significant changes in the mean of the LVEDd, LVVd, SV, CO, and EF variables. The changes in parameters like LVEDd, LVVd, SV, CO, and EF between two groups and in two measurement occasions were statistically significant. On the other hand, changes in LVESd, LVVs, and FS did not vary during the training between the two groups (Table 2).

**Table 2:** Repeated measures analysis (ANOVA) of variance results for echocardiographic parameters.

Variable	Time	Group	Time × group	
	F (p-value)	F (p-value)	F	p-value
LVESd (cm)	1.39 (0.272)	5.85 (0.042)	0.89	0.373
LVEDd (cm)	33.33 (< 0.001)	0.14 (0.713)	37.93	< 0.001
LVVd (mL)	22.37 (0.003)	0.38 (0.559)	51.02	< 0.001
LVVs (mL)	4.29 (0.084)	1.70 (0.240)	1.07	0.341
SV (mL)	71.44 (< 0.001)	0.70 (0.434)	138.57	< 0.001
CO (mL/min)	69.26 (< 0.001)	0.17 (0.694)	102.53	< 0.001
FS (%)	5.51 (0.057)	8.02 (0.030)	5.39	0.059
EF (%)	17.64 (0.006)	3.66 (0.104)	29.18	0.002

Left ventricle during end-systole dimensions (LVESd, cm), left ventricle during end-diastole dimensions (LVEDd, cm), left ventricular end-systolic volume (LVVs, mL), left ventricular end-diastolic volume (LVVd, mL), stroke volume (SV, mL), cardiac outputs (CO, mL/min), fractional shortening (FS, %), ejection fraction (EF, %).

**Figure 2:**
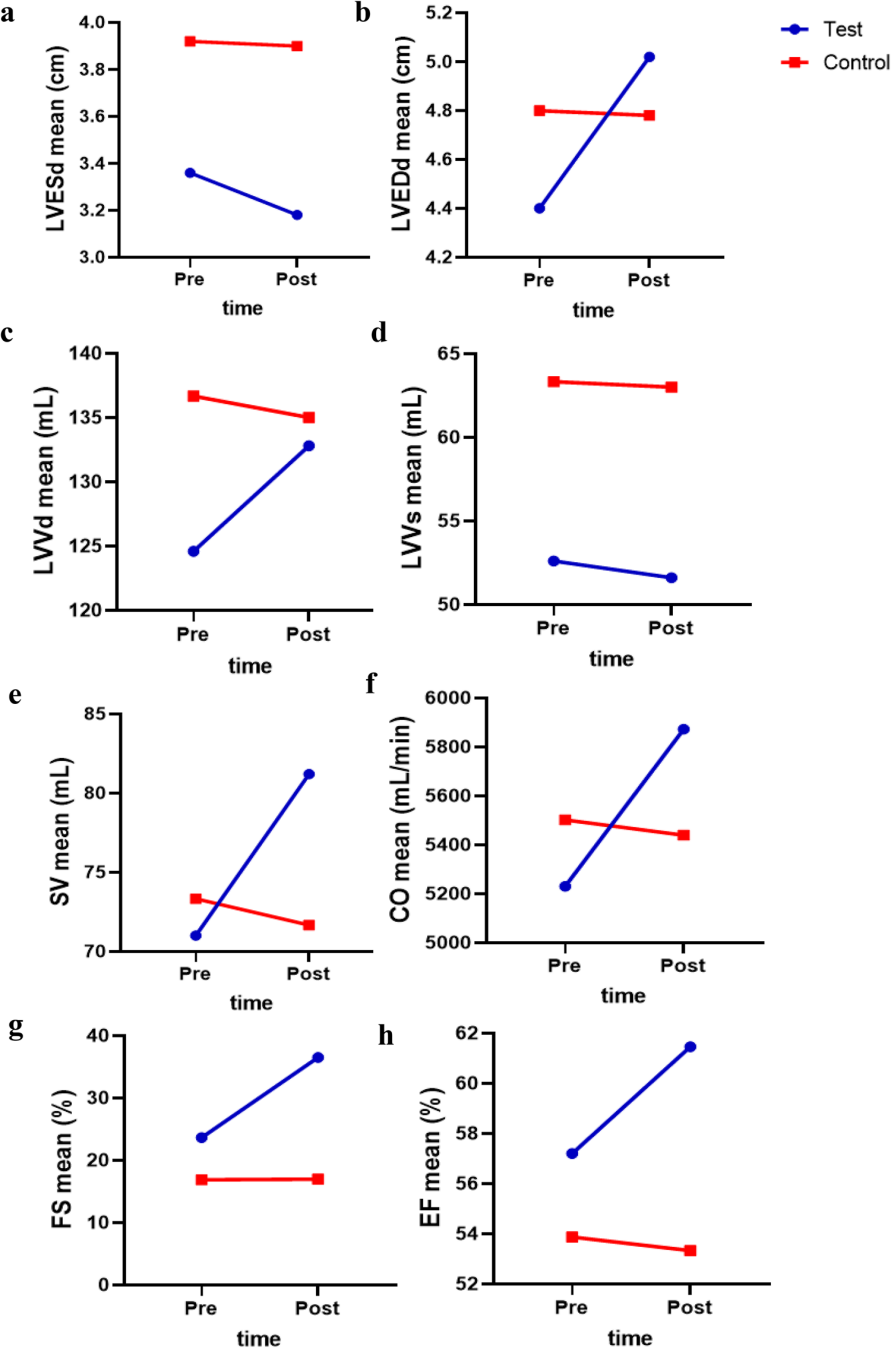
Trend graph of LVEDd, LVESD, LVVd, LVVs, SV, CO, FS, and EF. In the test group, the average of the variables LVEDd, LVVd, SV, and CO in the pre-test was lower than the average changes of the control group and in the post-test the average of the test group is higher. Left ventricle during end-systole dimensions (LVESd, cm), left ventricle during end-diastole dimensions (LVEDd, cm), left ventricular end-systolic volume (LVVs, mL), left ventricular end-diastolic volume (LVVd, mL), stroke volume (SV, mL), cardiac outputs (CO, mL/min), fractional shortening (FS, %), ejection fraction (EF, %).

To diagnose the changes in the variables at the pre and post-test and to check the type of change between the two groups, trend graphs as well as pairwise comparisons between the two groups. Based on this, Fig. 2 shows the changes between various times in separate groups.

According to figure, in the test group, the average of the variables LVEDd, LVVd, SV, and CO in the pre-test was lower than the average changes of the control group and in the post-test, the average of the test group was higher. To know the changes in separate times and groups, comparisons between two times and two groups were done, and the results were reported in the following tables.

### Functional resistance training effects on echocardiographic parameters in NAFLD patients

**Figure 3:**
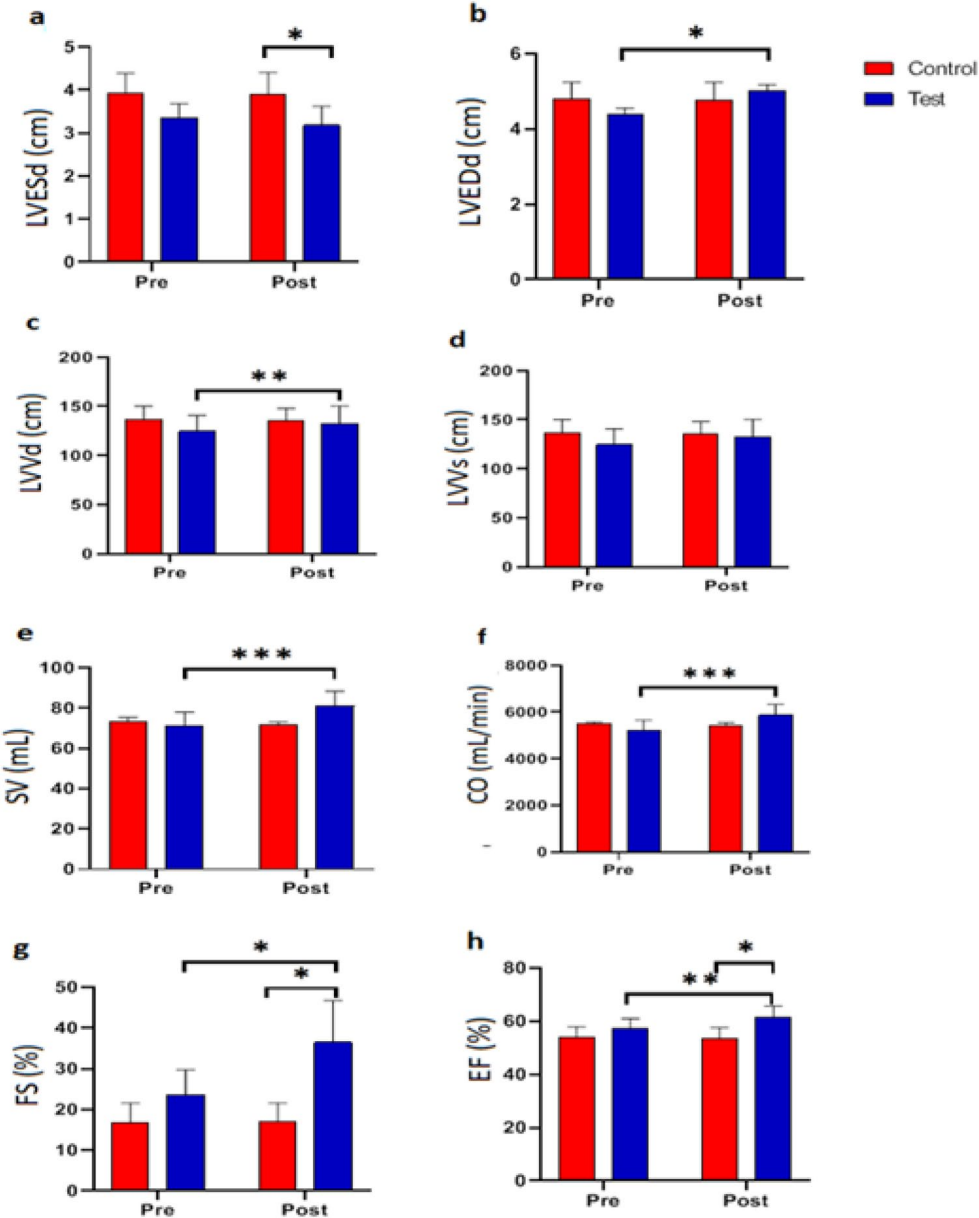
Effects of functional resistance training on echocardiographic parameters in the studied groups. The control group did not receive any intervention, but the test group did functional resistance training group 24 sessions over 8 weeks 3 sessions per week at a local gym. (**a**) Left ventricle during end-systole dimensions (LVES_d_, cm), (**b**) left ventricle during the end-diastole dimensions (LVED_d_, cm), (**c**) left ventricular end-systolic volume (LVV_s_, mL), (**d**) left ventricular end diastolic volume (LVV_d_, mL), (**e**) stroke volume (SV, mL), (**f**) cardiac outputs (CO, mL/min), (**g**) fractional shortening (FS, %), (**h**) ejection fraction (EF, %). Data are presented as means ± SD. *p-value < 0.05, **p-value < 0.001, ***p-value < 0.0001.

On the post-test, there was a significant difference between the average LVESd of the control and test groups (p-value = 0.04). In contrast, there was no significant difference between the average LVESd pre-test and post-test in the control and test groups. There was a significant difference in post-test scores between the control and test groups, indicating that functional resistance training significantly improved the systolic function of the heart (Table 3 and Fig. 3a.).

**Table 3:** Functional resistance training on the echocardiographic parameters.

Indicators	(n = 8)	Pre-test	Post-test	p-value/(post)	Figure 3
LVESd (cm)	Control group	3.92 ± 0.46	3.90 ± 0.50	> 0.05	a
	Test group	3.36 ± 0.32	3.18 ± 0.44	0.338	
	p-value	0.062	0.044		
LVEDd (cm)	Control group	4.80 ± 0.45	4.78 ± 0.46	0.37	b
	Test group	4.40 ± 0.16	5.02 ± 0.16	0.04	
	p-value	0.118	0.327		
LVVd (mL)	Control group	136.67 ± 13.58	135.00 ± 13.00	0.130	c
	Test group	124.60 ± 16.13	132.80 ± 17.70	0.001	
	p-value	0.322	0.859		
LVVS (mL)	Control group	63.33 ± 11.55	63.00 ± 11.27	0.423	d
	Test group	52.60 ± 11.78	51.60 ± 11.72	0.089	
	p-value	0.256	0.226		
SV (mL)	Control group	73.33 ± 2.08	71.67 ± 1.53	0.367	e
	Test group	71.00 ± 7.00	81.20 ± 7.26	0.004	
	p-value	0.604	0.072		
CO (mL/min)	Control group	5502.20 ± 65.46	5439.40 ± 106.37	0.208	f
	Test group	5230.20 ± 422.33	5872.60 ± 456.55	< 0.001	
	p-value	0.192	0.073		
FS (%)	Control group	16.90 ± 4.66	16.97 ± 4.63	0.959	g
	Test group	23.64 ± 6.13	36.50 ± 10.23	0.035	
	p-value	0.155	0.022		
EF (%)	Control group	53.87 ± 4.10	53.33 ± 4.30	0.150	h
	Test group	57.20 ± 3.90	61.46 ± 4.32	0.003	
	p-value	0.294	0.042	–-	

Left ventricle during end-systole dimensions (LVESd, cm), left ventricle during end-diastole dimensions (LVEDd, cm), left ventricular end-systolic volume (LVVs, mL), left ventricular end-diastolic volume (LVVd, mL), stroke volume (SV, mL), cardiac outputs (CO, mL/min), fractional shortening (FS, %), ejection fraction (EF, %).

Data are presented as means ± SD.

The difference in pre- and post-test mean LVEDd between the control and test groups was not statistically significant. In addition, there was no difference in the average diastolic function of the heart between the pre-test and post-test in the control group, whereas this difference was significant (p-value = 0.04) in the test group. According to Fig. 3, functional resistance training had a significant impact on the diastolic function of the heart in patients with non-alcoholic fatty liver (Table 3 and Fig. 3b).

The assumption that the pre-test and post-test LVVd levels were identical was not significant. The average value of the LVVs variable did not differ significantly between the control and test groups in either the pre-test or the post-test. In addition, there was no significant difference between the pre-test and post-test in the control group, whereas the difference in the average LVVd between the pre-test and post-test in the test group was significant (p-value = 0.001) (Table 3 and Fig. 3c). The effect of functional resistance training on LVVs was insignificant (Table 3 and Fig. 3d).

Significantly (p-value = 0.001), the supposition of mean SV equality between the pre-test and post-test in the test group is rejected. Consequently, it can be stated that resistance functional training had a substantial impact on SV (Table 3 and Fig. 3e).

The average CO difference between the pre-test and post-test groups in the test group was statistically significant (p-value = 0.001), indicating that functional resistance training had a significant effect on the CO variable (Table 3 and Fig. 3f).

At the pre-test, there was no significant difference in the average FS between the test and control groups; however, at the post-test, there was a significant difference (p-value = 0.01). In addition, the mean difference between the pre-test and post-test was not significant in the control group, whereas it was significant in the test group (p-value = 0.03) (Table 3 and Fig. 3g).

Before the post-test, there was no significant difference in the average EF between the two test and control groups, but after the post-test, there was a significant difference (p-value = 0.04). In addition, the mean difference between the pre-test and post-test in the control group was not significant, whereas this difference was significant in the test group (p-value = 0/004) (Table 3 and Fig. 3h). The control group did not receive any intervention, but the test group did functional resistance training group 24 sessions over 8 weeks three sessions per week at a local gym.

### Functional resistance training affects the trends of change in Hs-CRP, FibroScan, and liver enzymes parameters

**Figure 4:**
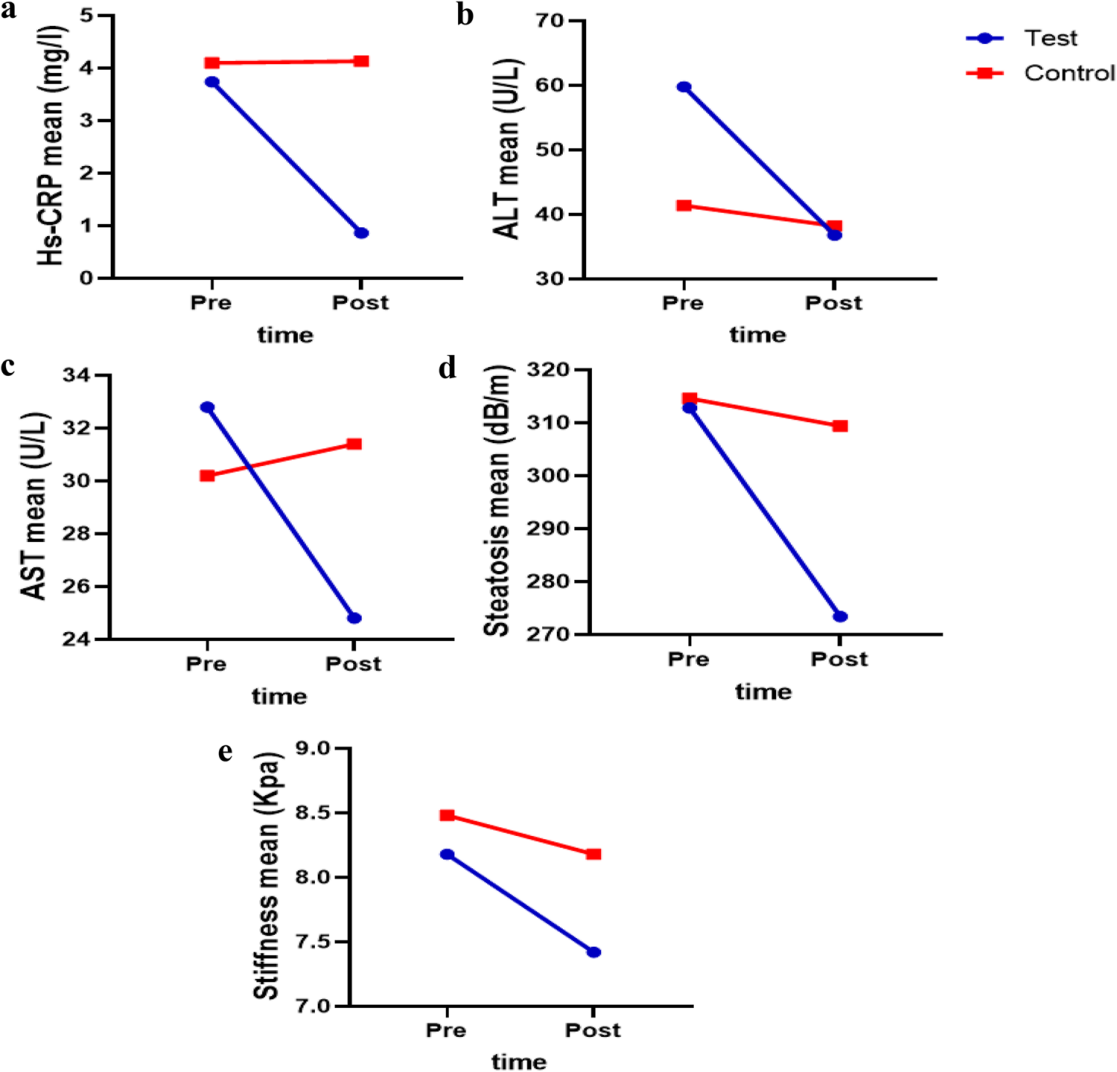
Trend graph of Hs-CRP, FibroScan, and liver enzymes. C-reactive protein (Hs-CRP, mg/L), alanine transaminase (ALT, U/L), aspartate aminotransferase (AST, U/L), steatosis (dp/m) and stiffness (kilopascal, kPa). According to the figure, the rate change of Hs-CRP in the control group was constant over time, but in the test group, a significant decrease was observed during times. The average values of liver enzymes in the pre-test in the test group are higher than the control group, but in the post-test, the average values are significantly lower than the control group. The average values of steatosis and stiffness in both groups at both times were lower in the test group, but they have a more severe decreasing trend.

Based on the results of the repeated measures ANOVA, the significance in time means that the change of time from the pre- to the post-test caused significant changes in the mean of the HS-CRP, ALT, AST, steatosis, and stiffness variables. The changes in parameters including HS-CRP, ALT, AST, and Steatosis, between two groups and in two measurement occasions are statistically significant (Table 4, Fig. 4).

**Table 4:** Repeated measures analysis of variance (ANOVA).

Variable	Time	Group	Time × group	
	F (p-value)	F (p-value)	F	p-value
Hs-CRP (mg/L)	43.11 (< 0.001)	80.54 (< 0.001)	45.19	< 0.001
ALT (U/L)	30.05 (0.001)	0.86 (0.381)	17.16	0.003
AST (U/L)	6.76 (0.032)	0.55 (0.480)	12.37	0.008
Steatosis (dB/m)	11.46 (0.010)	1.54 (0.250)	6.74	0.032
Stiffness (kPa)	26.64 (0.001)	1.61 (0.239)	4.64	0.063

Results for Hs-CRP, FibroScan, and liver enzyme parameters in the studied groups. C-reactive protein (Hs-CRP, mg/L), alanine transaminase (ALT, U/L), aspartate aminotransferase (AST, U/L), steatosis (dp/m) and stiffness (kiloPascal, kPa). To diagnose the changes in the variables at the pre and post-test and to check the type of change between the two groups, we used trend graphs as well as pairwise comparisons between the two groups.

### Functional resistance training effects on Hs-CRP, FibroScan, and liver enzymes in NAFLD patients

**Figure 5:**
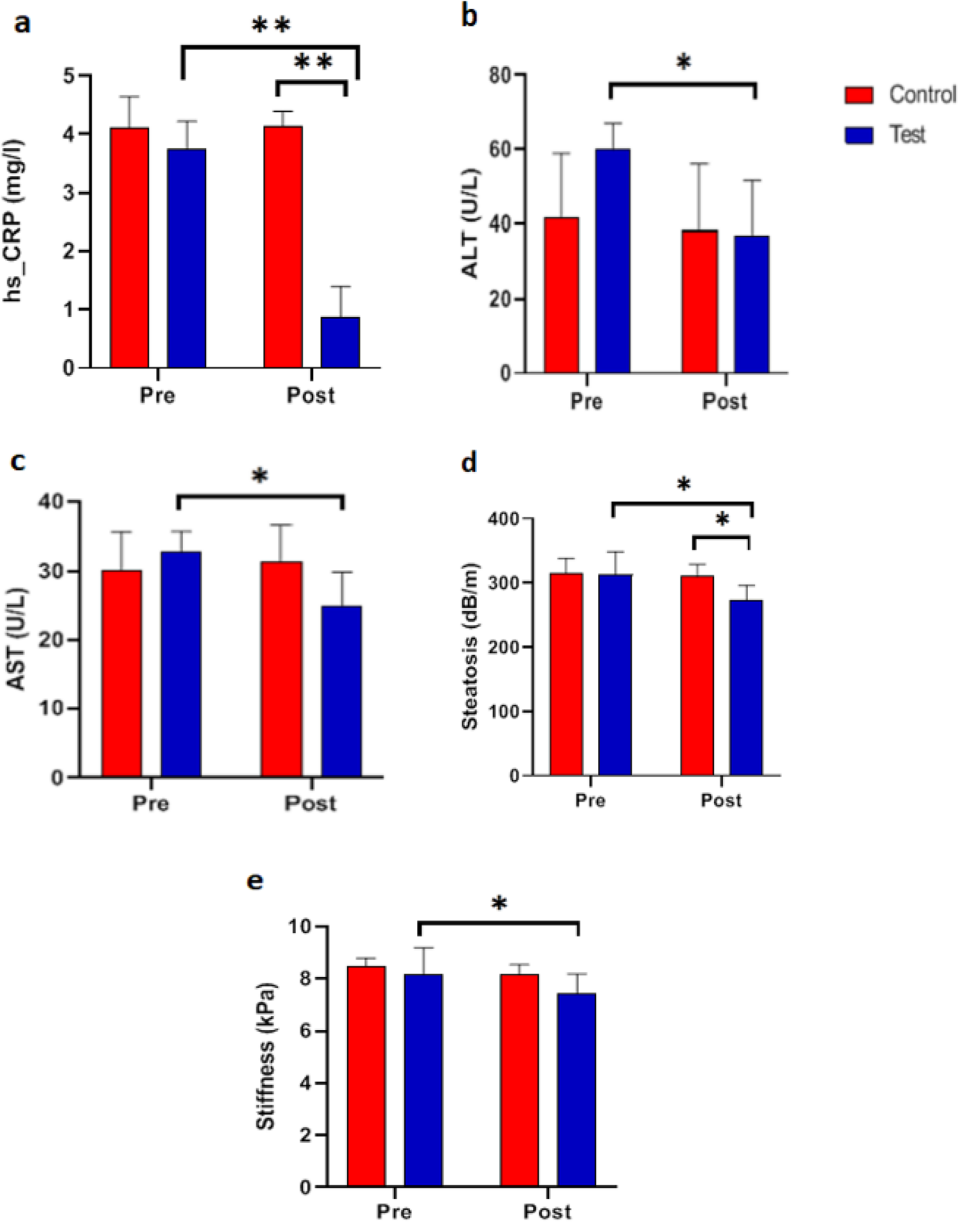
Effects of functional resistance training on Hs-CRP, FibroScan imaging, and liver enzymes in studied groups**.** The control group did not receive any intervention, but the test group did functional resistance training group 24 sessions over 8 weeks three sessions per week at a local gym. (**a**) High-sensitivity C-reactive protein (Hs-CRP, mg/L), (**b**) alanine transaminase (ALT, U/L), (**c**) aspartate aminotransferase (AST, U/L), (**d**) steatosis (dp/m) and (**e**) stiffness (kilopascal, kPa). Data are presented as means ± SD. *p-value < 0.05, **p-value < 0.001, *p-value < 0.05, **p-value < 0.001.

As shown in Table 5, the difference in mean Hs-CRP levels between the control and test groups after the test was statistically significant (p = 0.008), whereas only the test group showed a significant difference between pre-test and post-test levels (p = 0.001) (Table 5 and Fig. 5a). Based on the graph, it is evident that functional resistance training in the test group had a significant effect on the serum level of acute phase reactive protein at the level of 0.01 and that the difference between the control and test groups on the post-test was also significant.

**Table 5:** Functional resistance training on the FibroScan examination and liver function.

Indicators	(n = 8)	Pre-test	Post-test	p-value/(post)	Figure 5
Hs-CRP (mg/L)	Control group	4.10 ± 0.54	4.13 ± 0.26	0.911	a
	Test group	3.74 ± 0.48	0.86 ± 0.53	0.001	
	p-value	0.300	< 0.001		
ALT (U/L)	Control group	41.40 ± 17.53	38.20 ± 17.85	0.061	b
	Test group	59.80 ± 7.15	36.80 ± 14.82	0.008	
	p-value	0.062	0.896	–-	
AST (U/L)	Control group	30.20 ± 5.40	31.40 ± 5.18	0.109	c
	Test group	32.80 ± 2.86	24.80 ± 5.07	0.035	
	p-value	0.370	0.076		
Steatosis (dB/m)	Control group	314.60 ± 23.67	309.40 ± 19.36	0.106	d
	Test group	312.80 ± 35.88	273.40 ± 23.10	0.038	
	p-value	0.928	0.028		
Stiffness (kPa)	Control group	8.48 ± 0.33	8.18 ± 0.36	0.083	e
	Test group	8.18 ± 1.01	7.42 ± 0.77	0.011	
	p-value	0.310	0.080		

The control group did not receive any intervention, but the test group did functional resistance training group 24 sessions over 8 weeks three sessions per week at a local gym. High-sensitivity C-reactive protein (Hs-CRP, mg/L), alanine transaminase (ALT, U/L), aspartate aminotransferase (AST, U/L), steatosis (dp/m) and stiffness (kilopascal, kPa). Data are presented as means ± SD.

As can be seen, neither the pre-test nor the post-test means for the liver enzyme ALT differed significantly between the two groups. Alternatively, the results of the paired t-test indicated that in the control group, there is no significant difference between the pre-test and post-test levels of each liver enzyme ALT, whereas, in the test group, there is a significant difference (p = 0.008) (Table 5 and Fig. 5b). The graph demonstrates that the assumption of the equality of ALT average between the pre-test and post-test in the test group is rejected, indicating that functional resistance training has a significant effect on the serum level of liver enzyme ALT in patients with non-alcoholic fatty liver disease.

As can be seen, neither the pre-examination nor the post-examination means levels of the liver enzyme AST differed significantly between the two groups. In contrast, the results indicated that the difference in AST between the pre-test and post-test in the control group is not significant, whereas, in the test group, the difference in these enzymes between the pre-test and post-test is significant (p = 0.03) (Table 5 and Fig. 5c). It can be seen from the graph that the assumption of AST equality between the pre-test and post-test in the test group is rejected, indicating that functional resistance training had a significant effect on the serum.

Before the test, the difference in average steatosis between the control and test groups was not significant; however, this difference was significant after the test (p = 0.03) (Table 5 and Fig. 5d). In contrast, the difference in liver fat between the pre-test and post-test in the control group was not significant, whereas this difference was significant (p = 0.04) in the test group. At the 0.05 significance level, the assumption of average hepatic fat equality in the test group is rejected. In addition, there is a significant difference between the mean liver fat before and after the test in the test group.

The difference between the mean levels of liver fibrosis in the control and test groups was not significant before and after the evaluation. The difference between pre-test and post-test means for liver fibrosis in the test group is significant at the 0.05 level (Table 5 and Fig. 5e). It is evident from the graph that functional resistance training was responsible for the change in post-test liver fibrosis in the test group.

### Functional resistance training affects the trends of change in the biochemical parameters

**Figure 6:**
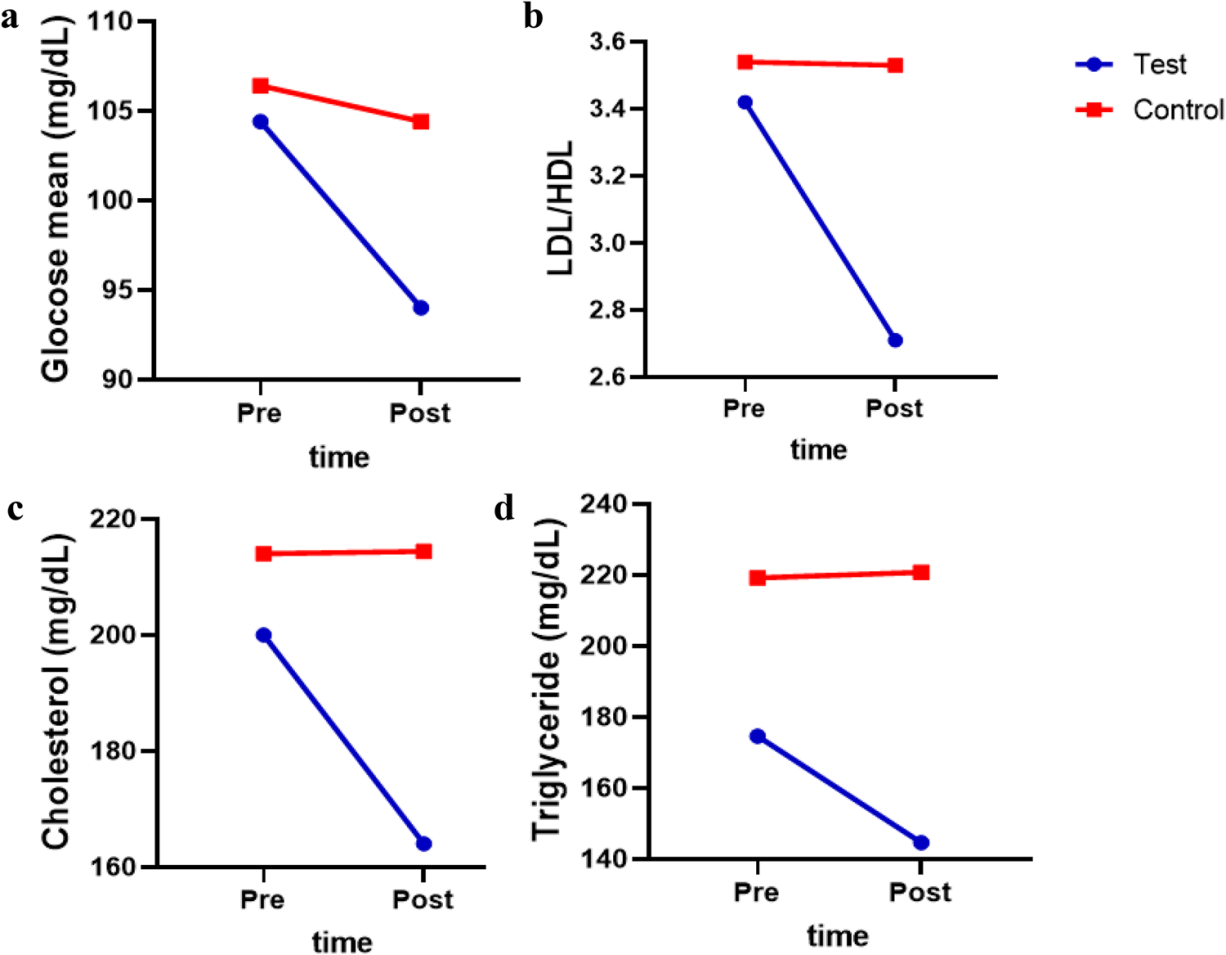
Trend graph of the biochemical parameters. High-density lipoprotein (HDL)/low-density lipoprotein (LDL). Based on the figure, the biochemical parameters have decreased during two measurements in test groups, but the trend of changes for cholesterol and triglyceride variables was constant in the control group. Also, all the values for the test group in the pre and post-test were lower than the control group.

The results of the repeated measures ANOVA showed variables like TG and Chol change between two groups in pre- and post-measurement were statistically significant (Table 6). The trend graphics for these variables is shown in Fig. 6.

**Table 6:** Repeated measures analysis (ANOVA) of variance results for biochemical.

Variable	Time	Group	Time × group	
	F (p-value)	F (p-value)	F	p-value
Glucose (mg/dL)	9.23 (0.016)	0.53 (0.486)	4.23	0.074
LDL/HDL	5.56 (0.046)	1.91 (0.205)	5.14	0.053
TG (mg/dL)	10.90 (0.011)	3.84 (0.099)	13.50	0.006
Chol (mg/dL)	20.13 (0.002)	3.70 (0.091)	21.04	0.002

Glucose (mg/dL), high-density lipoprotein (HDL)/low-density lipoprotein (LDL), triglyceride (TG, mg/dL), and cholesterol (Chol, mg/dL).

### The effects of functional resistance training on the biochemical parameters of study groups

**Figure 7:**
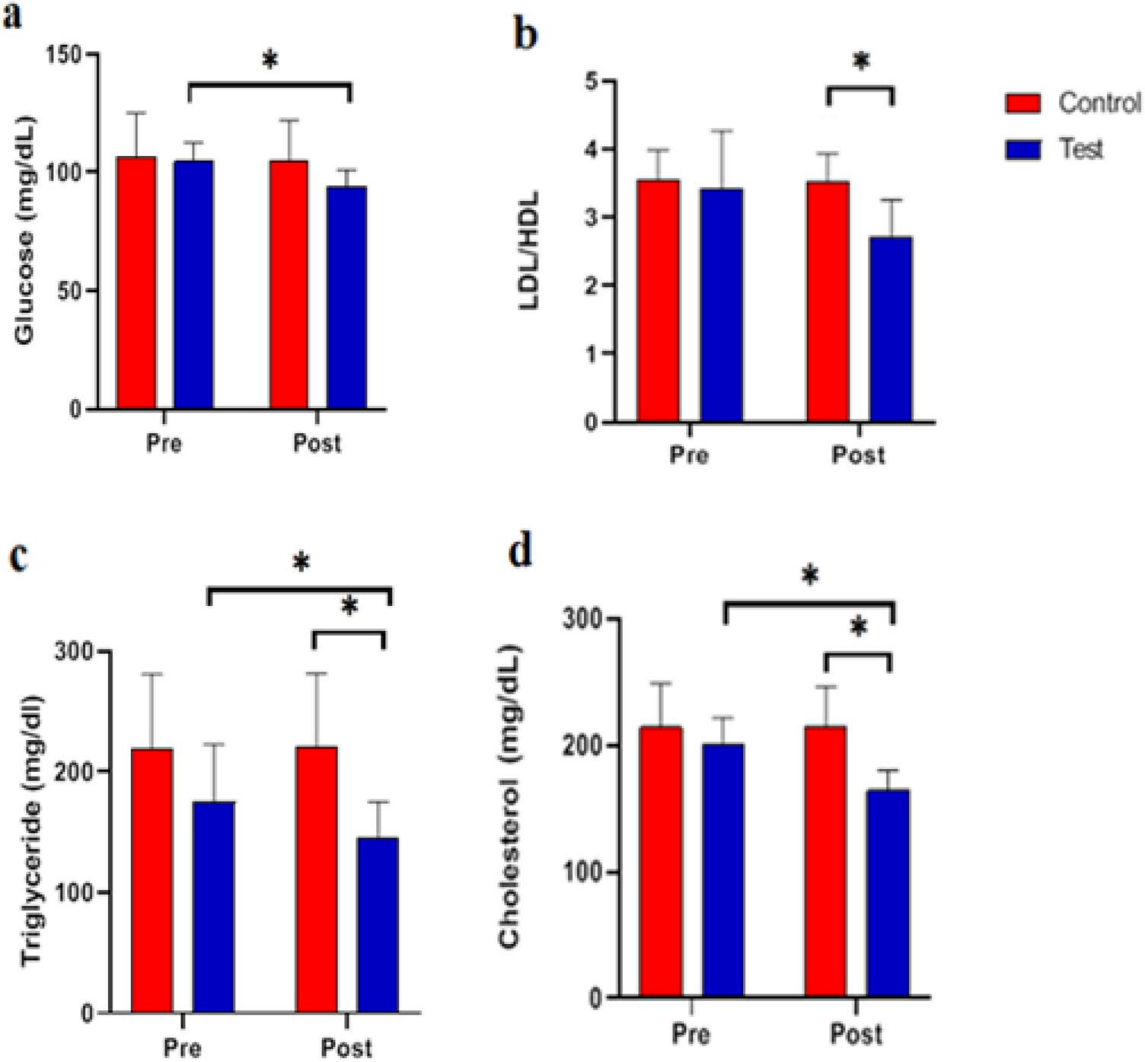
Effects of functional resistance training on Biochemical parameters in studies groups. In this study, the control group did not receive any intervention, but the test group did functional resistance training group 24 sessions over 8 weeks three sessions per week at a local gym. (**a**) Glucose (mg/dL), (**b**) high-density lipoprotein (HDL)/low-density lipoprotein (LDL), (**c**) cholesterol (mg/dL) (**d**) triglyceride (mg/dL). Data are presented as means ± SD. *p-value < 0.05.

Before and after the test, there was no significant difference in the variable value of glucose between the control and test groups. The difference between pre-test and post-test average glucose levels in the control group is not statistically significant (p-value 0.05), whereas this difference was significant in the test group (p-value 0.05) the test group's functional resistance training led to a significant difference in post-test blood glucose levels (p = 0.04) (Table 7 and Fig. 7a). The difference in mean LDL/HDL between the control and test groups was statistically significant in the post-test. In addition, there was no significant difference between the pre-test and post-test means of LDL/HDL in the control and test groups (Table 7 and Fig. 7b).

**Table 7:** Functional resistance training on biochemical tests.

Indicators	Groups Control group (n = 8) Test group (n = 8)	Pre-test	Post-test	p-value (post)	Figure 7
Glucose (mg/dL)	Control group	106.40 ± 18.51	104.40 ± 17.44	0.426	a
	Test group	104.40 ± 8.08	94.00 ± 7.10	0.038	
	p-value (in each group)	0.830	0.269		
LDL/HDL	Control group	3.54 ± 0.44	3.53 ± 0.40	0.764	b
	Test group	3.42 ± 0.84	2.71 ± 0.54	0.080	
	p-value (in each group)	0.789	0.028		
TG (mg/dL)	Control group	219.20 ± 61.38	220.80 ± 60.29	0.347	c
	Test group	174.60 ± 48.15	144.60 ± 30.64	0.024	
	p-value (in each group)	0.237	0.036		
Chol (mg/dL)	Control group	214.00 ± 34.58	214.40 ± 32.16	0.772	d
	Test group	200.00 ± 21.73	164.00 ± 16.00	0.010	
	p-value (in each group)	0.465	0.014		

The control group did not receive any intervention, but the test group did functional resistance training group 24 sessions over 8 weeks three sessions per week at a local gym. Glucose (mg/dL), high-density lipoprotein (HDL)/low-density lipoprotein (LDL), triglyceride (TG, mg/dL), and cholesterol (Chol, mg/dL).

Data are presented as means ± SD.

There was no significant difference between the control and test groups on the pre-test. In addition, the average of these variables between the pre-test and post-test did not differ significantly in the control group, whereas the average triglyceride did differ significantly (p = 0.02) in the test group. At the post-test, the average triglyceride difference between the control and test groups was statistically significant (p = 0.04) (Table 7 and Fig. 7c). The number of triglycerides is significantly influenced by functional resistance training.

There was no significant difference in cholesterol levels between the control and test groups in the pre-test. In contrast, there was a significant difference between pre-test and post-test cholesterol levels between the control and test groups (p = 0.01). In addition, the difference in post-test mean cholesterol levels between the control and test groups was statistically significant (p = 0.05) (Table 7 and Fig. 7d).

## Discussion

This is one of the first perusals to assess the effects of functional resistance training on the structure and function of the heart and liver in patients with Non-Alcoholic Fatty Liver**.** In the current study, functional resistance training resulted in improving EF and FS in the heart. On the other hand, the reduction of liver stiffness, and steatosis, as well as the modification of ALT and AST enzymes, improved liver function in non-alcoholic fatty liver patients.

Some previous studies indicated that there is insufficient evidence to demonstrate that functional training enhances anthropometric and functional measures more effectively than conventional resistance training^12^. But in 2009, Kibele and colleagues found that strength and other performance evaluations, including dynamic balance and shuttle running, did not differ significantly compared to a more traditional resistance training program^23^.

Considering all functional resistance training can be performed at home with minimal facilities, cost, and physical space, and due to the health conditions of the society in terms of quarantine and traffic bans due to the spread of the Corona Virus Disease 19 (COVID-19) pandemic, it is likely that these exercises can perform additional functions. The individual improves the structure and function of the heart and liver in individuals with NAFLD.

The most prevalent chronic liver disease, NAFLD is characterized by fat accumulation in more than 5% of hepatocytes and a wide spectrum of liver injuries, including nonalcoholic fatty liver, nonalcoholic steatohepatitis, substantial fibrosis, and cirrhosis. NAFLD-related^1^. In the United States, NAFLD-related fibrosis is on the rise, which could lead to an increase in overall and cause-specific mortality^24^. A better comprehension of modifiable risk factors for NAFLD and significant fibrosis can have a substantial impact on reducing the disease's severity and associated costs. Given the close association between central obesity, type 2 diabetes, dyslipidemia, and the prevalence of NAFLD, and the role of exercise activities in reducing the incidence and complications of NAFLD, functional resistance training activity can improve complications caused by NAFLD^25^. After controlling for other confounding variables, previous research has confirmed an inverse relationship between physical activity and NAFLD prevalence^26^. In addition, a recent longitudinal study demonstrated the protective effect of physical activity (PA) at baseline on incident NAFLD and the protective effect of sustained or increased PA on the development of NAFLD independent of visceral adiposity and insulin resistance^27^. In addition, the difference between the average percentage of body fat in the pre-test and post-test in the control group was not significant, whereas this difference was significant in the test group; that is, the average percentage of body fat in the test group differs significantly between the pre-test and post-test (p-value = 0.03). Previous studies also demonstrated that resistance training improved the BMI of overweight and obese individuals^28–30^.

In patients with non-alcoholic fatty liver disease, functional resistance training has a considerable influence on diastolic and systolic heart function, LVESd, and LVEDd. The findings of this study are consistent with the findings of numerous earlier studies^31–34^. Despite a significant increase in LV mass, intense resistance training increases LV diastolic function at rest and during isometric exercise. In the present study, the pre-test and post-test mean values of the LV diastolic volume variable did not differ significantly in the control group, but in the test group, the results were significant (p-value = 0.001)^35^. In general, functional resistance training did not affect the LV systolic volume. Although a high heart rate during exercise increases the number of repetitions and cardiac output for the working muscles, it may also be considered a factor that decreases ventricular filling by shortening the diastolic period^36^. In addition, it is well known that high-intensity resistance training (RT) leads to concentric cardiac hypertrophy by increasing left ventricular wall thickness and cardiac cell diameter while leaving the internal cavity size unchanged^37^. Hearts that have been trained for endurance were subject to increased volume and pressure loading, resulting in myocardial alterations such as left ventricular dilatation and increased left ventricular mass. Meaningful increases in systolic and diastolic blood pressure are associated with resistance training^38^. In addition, functional resistance training had a significant impact on SV (p-value < 0.001). Resistance training increases muscle mass and strength, as well as cardiac filling volume, SV, exercise capacity, and CO^39, 40^. The mean CO difference between pre-and post-test groups in the test group was significant (p-value < 0.001), indicating that functional resistance training has a significant effect on the CO variable. Regular exercise augments venous return to the heart, thereby augmenting CO^41^. It has been shown that resistance training increases muscle strength, endurance, athletic performance, and physical performance in geriatric disease patients. Coronary artery disease and congestive heart failure resistance training can aid in the improvement of certain cardiovascular risk factors^42^. This index represents the EF, which is the left ventricle's systolic capacity. Evaluating the EF of the left ventricle using the total quantity of blood entering the left ventricle and the amount of blood remaining after one heartbeat^43, 44^. However, the average difference between the pre-test and post-test in the control group was not significant, whereas the average EF difference between the pre-test and post-test in the test group is significant (p-value < 0.003). Previous research indicates that exercise training increases EF and FS^45^. On the pre-test, there was no significant difference between the average FS of the two test and control groups, but on the post-test, there is a significant difference between the average FS of the two test and control groups (p-value = 0.01). In addition, the mean difference between the pre-test and post-test was not statistically significant in the control group, but it was in the test group (p-value = 0.03). The progression of NAFLD to NASH is partially attributable to inactivity. The likelihood of developing NAFLD increases by 4% for every hour per day of inactivity^46^. Recent research recommends at least 150 min per week of moderate-intensity activity or 75 min per week of vigorous-intensity activity for all individuals^47^. In a recent study of over 5,000 NAFLD patients by Kim et al., every 10 min of physical activity was associated with a 7% decrease in total mortality^48^. There is currently no accepted medication treatment for NAFLD and NASH, and the treatment principles include dietary and lifestyle modifications and exercise. Lifestyle modification necessitates a 5 to 10 percent weight reduction to repair the tissue structure in NASH^49^. Despite this, less than 10% of patients can lose this much weight^50^.

Randomized controlled trials were administered for 16 weeks to 18 patients with hepatic steatosis in research conducted by Sullivan et al. in 2020. Without any weight loss, hepatic steatosis was reduced by 10%, according to the results. While hepatic steatosis increased by 2% in the control group that received standard NAFLD counseling, hepatic steatosis decreased by 2% in the experimental group^51^. This study confirms our previous findings that functional resistance training reduces the quantity and severity of hepatic steatosis and fibrosis in NAFLD patients. In one study, Zhang et al. demonstrated that weight loss (3–6% of total body weight) is more likely than exercise intensity to reduce intrahepatic triglycerides than exercise intensity^52^.

In the present study, functional resistance training had a significant effect on the serum level of acute phase reactive protein in the test group (p-value = 0.001), and the difference between the control and test groups on the post-test was also significant (p-value = 0.008). Homocysteine and Hs-CRP levels in the serum are known risk factors for atherosclerosis and related cardiovascular diseases. Our research has reduced the serum concentration of Hs-CRP. These results indicate that aerobic and core exercise interventions effectively reduced serum inflammatory marker concentrations, even at modest levels^53^. Short-term and transient increases in serum CRP are caused by the exercise-induced acute phase response (APR), which is mediated by the cytokine system, particularly IL-6. There is a homeostatic anti-inflammatory APR after intense exercise, which may inhibit this response^54^. Chronic physical activity decreases resting CRP levels through multiple mechanisms, including decreased cytokine production by adipose tissue, skeletal muscle, endothelial mononuclear cells, and blood, enhanced endothelial function and insulin sensitivity, and an antioxidant effect^54^.

The test group's functional resistance training has led to a significant difference in post-test blood glucose levels (p-value = 0.04). In previous prospective studies involving healthy subjects^55^, subjects with impaired glucose tolerance^56^, and subjects with type 2 diabetes, resistance training improved glucose levels by 22–48%. This process is attributed to an increase in glucose's non-oxidative metabolism.

ALT and AST levels can remain elevated for at least 7 days following vigorous exercise. The greater the intensity and duration of the training, the greater the peak levels and the longer the levels remain elevated. Athletes without training will experience greater and lengthier increases than those with training. In the present study, functional resistance training had a significant effect on the serum levels of liver enzymes ALT and AST in nonalcoholic fatty liver disease patients (respectively p-value = 0.008 and 0.03)^57^.

The effects of exercise on endothelial function and inflammation have a positive impact on arterial rigidity^58^. Moreover, aerobic exercise reduces arterial stiffness in individuals^58^, whereas rigorous resistance training increases arterial stiffness^59^. It is evident from the diagram that functional resistance training has caused the change in liver fibrosis and steatosis in the test group following the test. Nonetheless, resistance training may also cause transient increases in arterial rigidity^60^. In addition, several chronic effects studies indicate that middle-aged men who engage in resistance training experience a significant increase in arterial rigidity^21^. Although age and hypertension are the two most important determinants of arterial rigidity, other factors also play a role. Large arteries stiffen and systolic pressure and pulse rise in the elderly hypertensive are due to wave reflection (a rising stiffness slope moving from heart to periphery)^61^.

In the development and progression of coronary artery disease, lipid and lipoprotein abnormalities play a crucial role. Improved serum lipid profile, blood pressure, and inflammatory markers, as well as a decreased risk of stroke, acute coronary syndrome, and overall cardiovascular mortality, are among the advantages. In addition, there is no significant difference between the pre-test and post-test means of LDL/HDL between the control and test groups, but Functional resistance training could reduce the ratio of LDL to HDL. One of the numerous advantages of strength training is that it can enhance cholesterol by decreasing LDL and increasing HDL^62^. Moreover, the difference between pre-test and post-test mean cholesterol levels in the test group was statistically significant (p-value = 0.01). In the test group, however, there was a significant difference between the pre-test and post-test mean levels of triglycerides, suggesting that functional resistance training impacts the variable level of triglycerides (p-value = 0.02). Several studies have demonstrated that exercise can enhance serum LDL-C, LDL-C, and TG levels, while other studies found no difference after exercise^63, 64^.

In conducting the current research, we were faced with limitations such as traffic prohibitions and strictures during the outbreak of the COVID-19 disease. Another limitation of the research was that the male operator was not able to perform the test on women due to considerations and national laws. Considering the positive effects observed from functional resistance training, it is suggested to investigate the effects of this training in the rehabilitation of patients with cardiovascular disease. It is also suggested to measure and analyze the level of arterial blood gases during the exercises.

## Conclusions

According to the findings, functional resistance training influences the structure and function of the heart.

In patients with nonalcoholic fatty liver disease. By investigating additional dimensions and conducting additional clinical and experimental studies, this method can be prescribed as a treatment for non-alcoholic fatty liver in physical therapy centers. Due to the nature of the exercises, it is also possible to maintain or improve flexibility through functional resistance training (multi-joint focusing on the full range of motion). This can be advantageous for older individuals, who are commonly characterized by poor or declining flexibility. In addition, many of the functional exercises used in this study can be performed in a variety of settings (gym, residence, travel), which may increase adherence (Supplementary Information).

### Supplementary Information

Supplementary Information.

## Data Availability

The data that support the findings of this study are available from the corresponding author upon reasonable request.

## Abbreviations

BMI: Body mass index
LVEDd: Left dilated ventricular end-diastolic diameter
LVESd: Left ventricle end-systolic dimension
EF: Ejection fraction
LVVd: Left ventricular (LV) volumes at end-diastole
SV: Stroke volume
CO: Cardiac output
FS: Fractional shortening
Hs-CRP: High-sensitivity C-reactive protein
ALT: Alanine transaminase
AST: Aspartate aminotransferase
TG: Triglycerides
Chol: Cholesterol
LVVs: Left ventricular volumes
LDL: Low-density lipoprotein
HDL: High-density lipoprotein
Hcy: Homocysteine
