# Supplementary material for: Effect of functional resistance training on the structure and function of the heart and liver in patients with non-alcoholic fatty liver
Source: Sci Rep. 2023 Sep 19;13:15475. doi: 10.1038/s41598-023-42687-w (PMC10509216; doi:10.1038/s41598-023-42687-w)
Supplement: Supplementary file 1 — Supplementary Information. [file 41598_2023_42687_MOESM1_ESM.docx]

**Supplementary data**

**Effect of Functional Resistance Training on the Structure and Function of the Heart and Liver in Patients with Non-Alcoholic Fatty Liver**

Ramin Jafarikhah^1^, Arsalan Damirchi^1*^, Farhad Rahmani nia^1^, Seyyed Mohammad Taghi Razavi-Toosi^2,3^, Afshin Shafaghi^4^, Mostafa Asadian^4^

^1^ Department of Sports Sciences, University of Guilan, Rasht, Iran

^2^ Medical Biotechnology Research Center, School of Paramedicine, Guilan University of Medical Sciences, Rasht, Iran

^3^ Cardiovascular Diseases Research Center, Department of Cardiology, Heshmat Hospital, School of Medicine, Guilan University of Medical Sciences, Rasht, Iran

^4^ Gastroenterologist Hepatologist, GI Cancer Screening and Preventing Research Center (GCSPRC), Guilan University of Medical Sciences, Rasht, Iran

^*^*Corresponding author:* Arsalan Damirchi

*Tel* :+98-911-1313084

*E-mail address:* [damirchiarsalan08@gmail.com](mailto:damirchiarsalan08@gmail.com); [damirchi@guilan.ac.ir](mailto:damirchi@guilan.ac.ir) (Arsalan Damirchi)

**1. Echocardiography Evaluation of the Studied Groups in NAFLD Patients**

Using echocardiography, the cardiac function was evaluated (SONOSCAPE P15 device). Transthoracic echocardiography was used according to the standard method to evaluate cardiac function [S1]. In both participant groups, identical echocardiograms were undertaken. Electrocardiography was performed concurrently with echocardiography to examine the heart's rhythm and rate. A specialist doctor who was unaware of the treatment protocol administered echocardiography examinations. Initially, two-dimensional images were captured from the perspective of the left ventricle's short axis in the mid-parasternal section. Then, M-mode images were acquired from the parasternal 4 long-axis levels of the papillary muscles of the left ventricle to determine the following parameters.

Dimensions of the left ventricle after systole (LVESd, cm) (a)

Dimensions of the left ventricle after diastole (LVEDd, cm) (b)

Left Ventricular end-systolic volume (LVV_s_ & mL) = 1.047 × (LVS_d_) ^3^  (c)

Left Ventricular end diastolic volume (LVV_d_ & mL) = 1.047 × (LVD_d_) ^3^ (d)

Stroke Volume (SV & mL) = Diastolic volume – Systolic volume (e)

Cardiac Outputs (CO & mL/min) = EF × Heart Rate (f)

The left ventricle's fractional shortening (FS) (g) and ejection fraction (EF) (h) were calculated using the following formulas [S2].

FS (%) = 100 × [(LVD_d_-LVS_d_)/LVD_d_]

EF (%) = 100 × [(LVD_d_^3^-LVS_d_)^3^/LVD_d_^3^]

2. **Assessment of Steatosis and Stiffness in the Examined Categories**

In clinical practice, transient elastography has evolved into a precise method and noninvasive instrument for evaluating hepatic steatosis and in terms of hepatic fibrosis. This investigation utilized transient elastography by previously accepted procedures. Liver *steatosis and stiffness* were assessed by (Fibro Touch- FT 100- ) Subjectively, steatosis can be categorized as minimal, moderate, or severe. Based on previous studies, a minor modification was made to the assessment and grading of liver stiffness and steatosis. [S3]

**
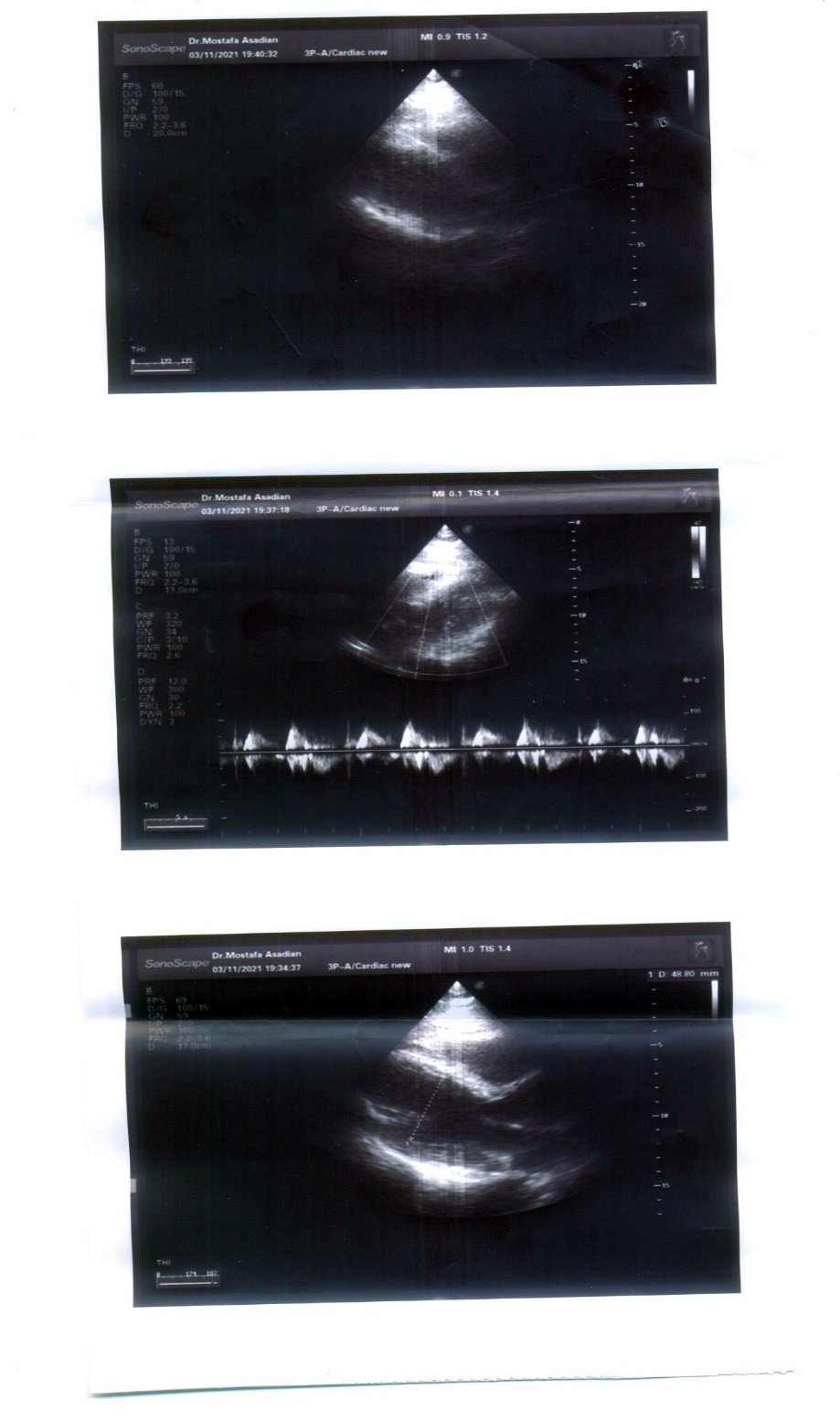
**

**Figure S1** (Echocardiography Pre- test for intervention patient NO.1)

**
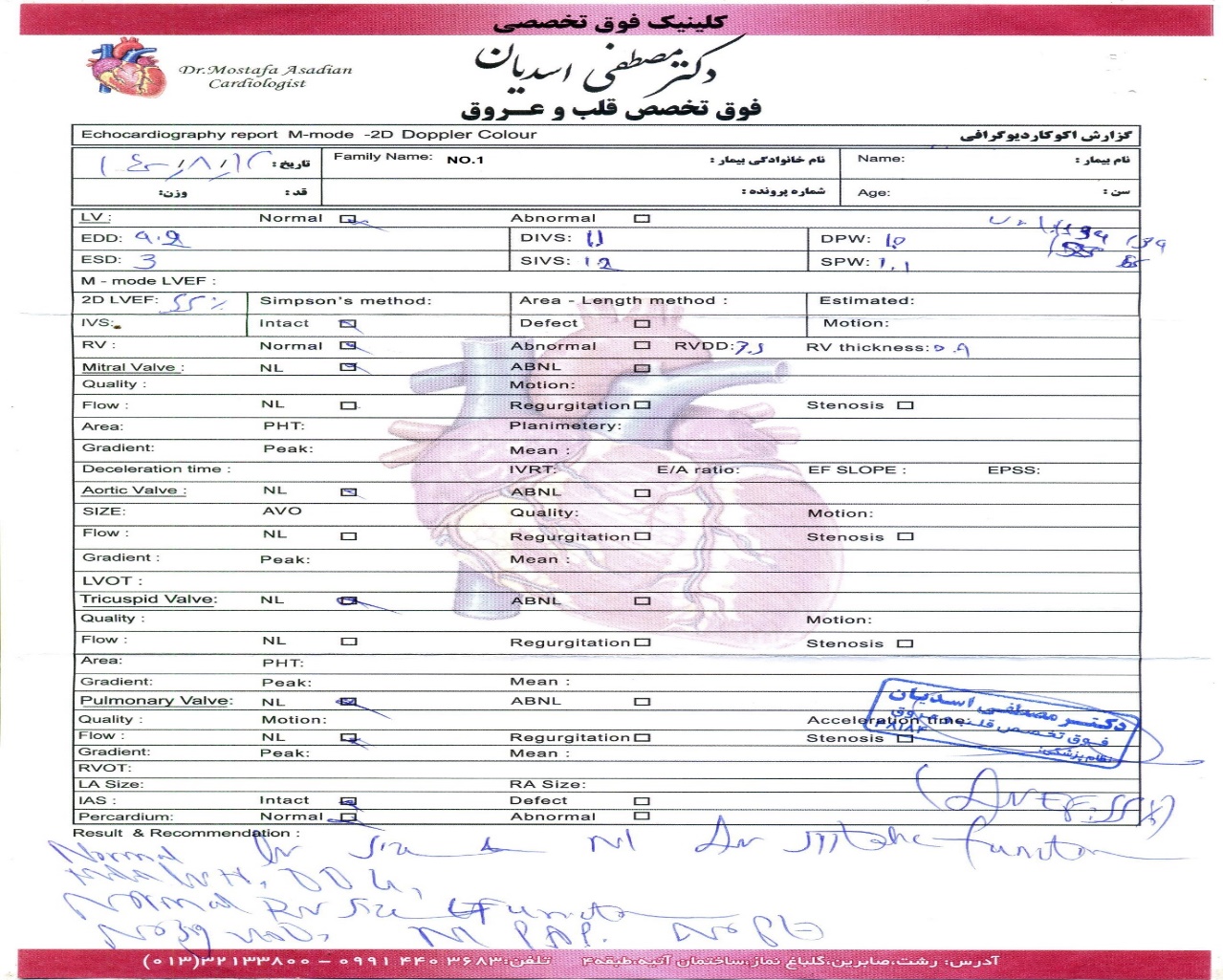
**

**Figure S2** (Echocardiography Pre- test for intervention patient NO.1)

**
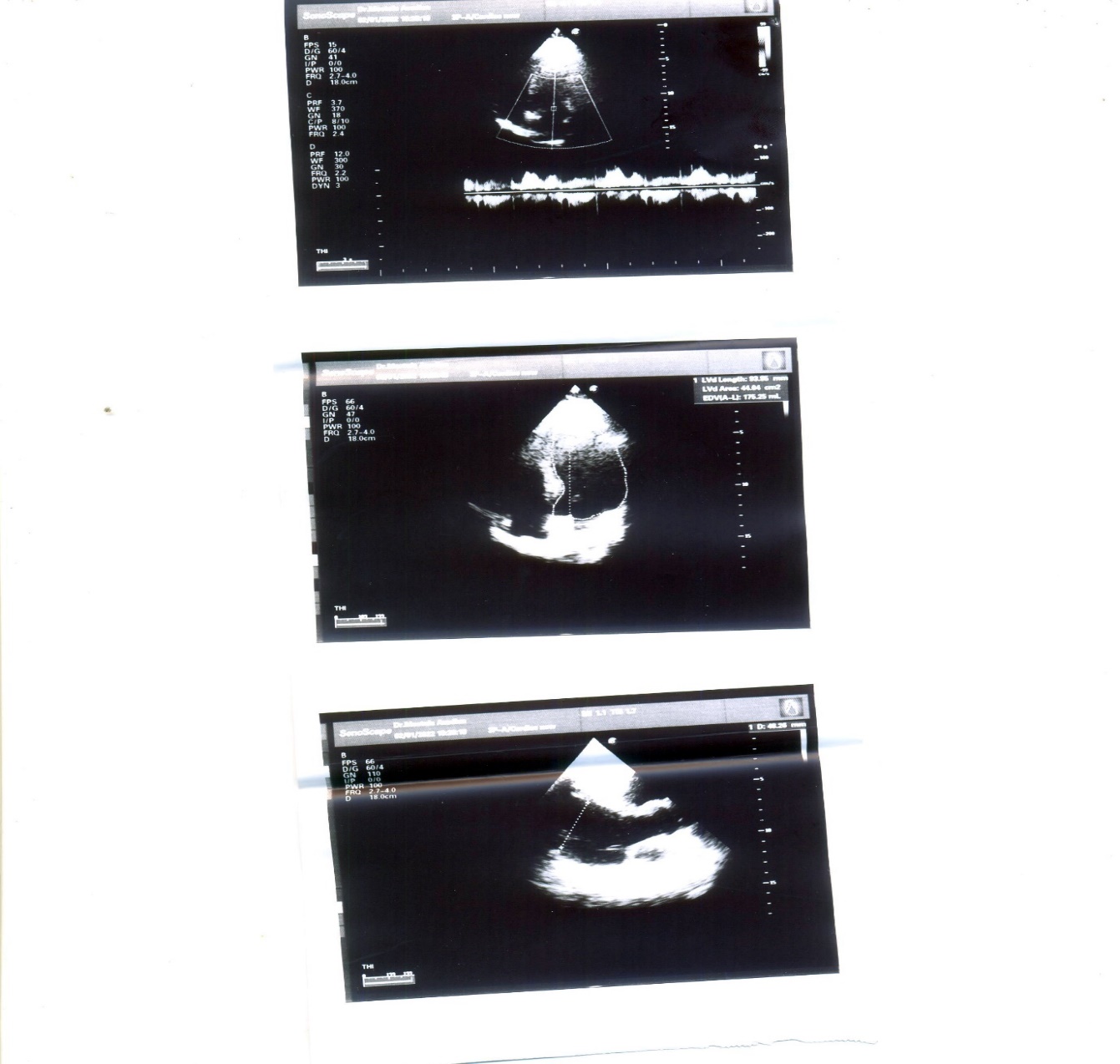
**

**Figure S3** (Echocardiography Post- test for intervention patient NO.1)

**
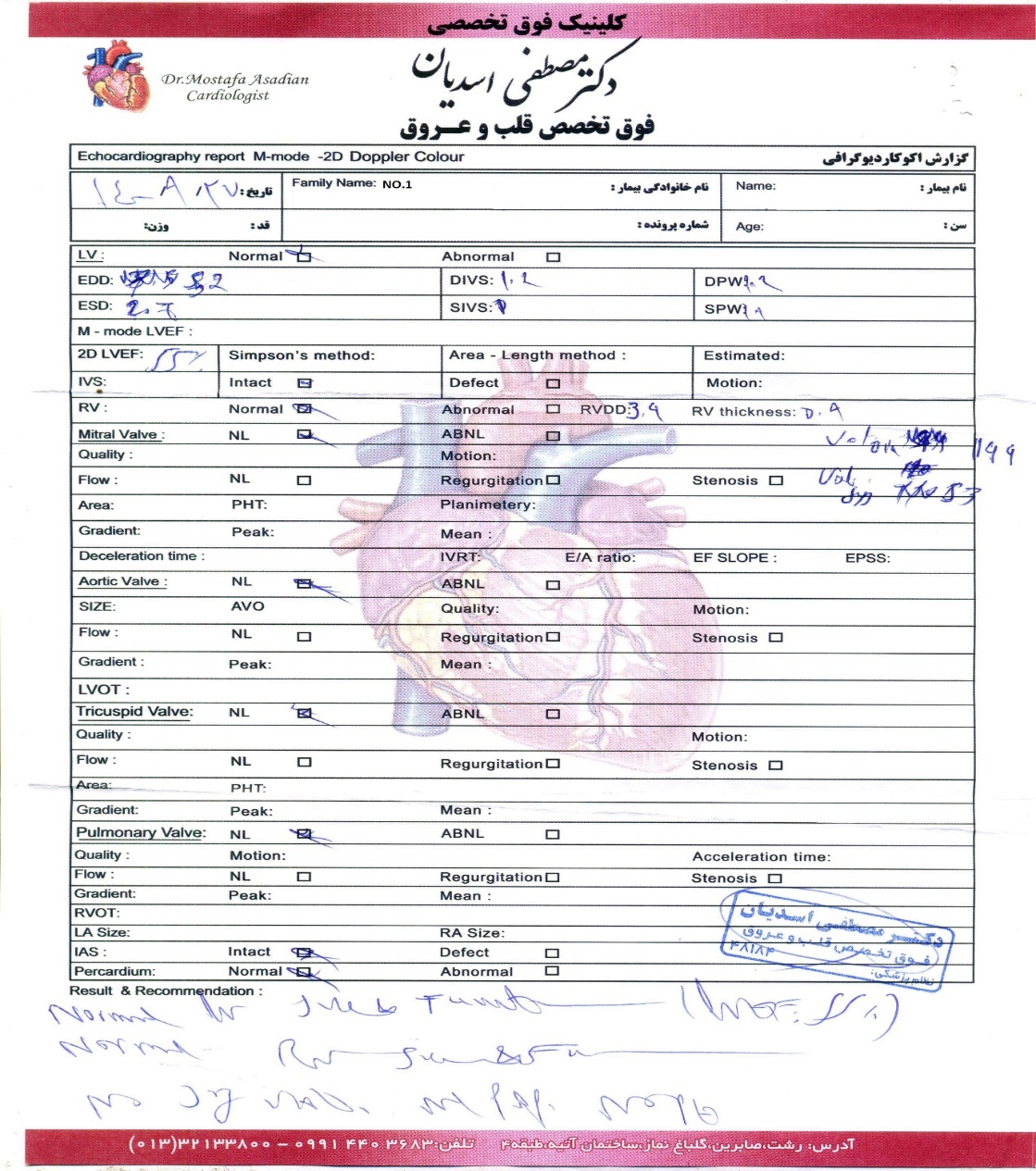
**

**Figure S4** (Echocardiography Post-test for intervention patient NO.1)

**
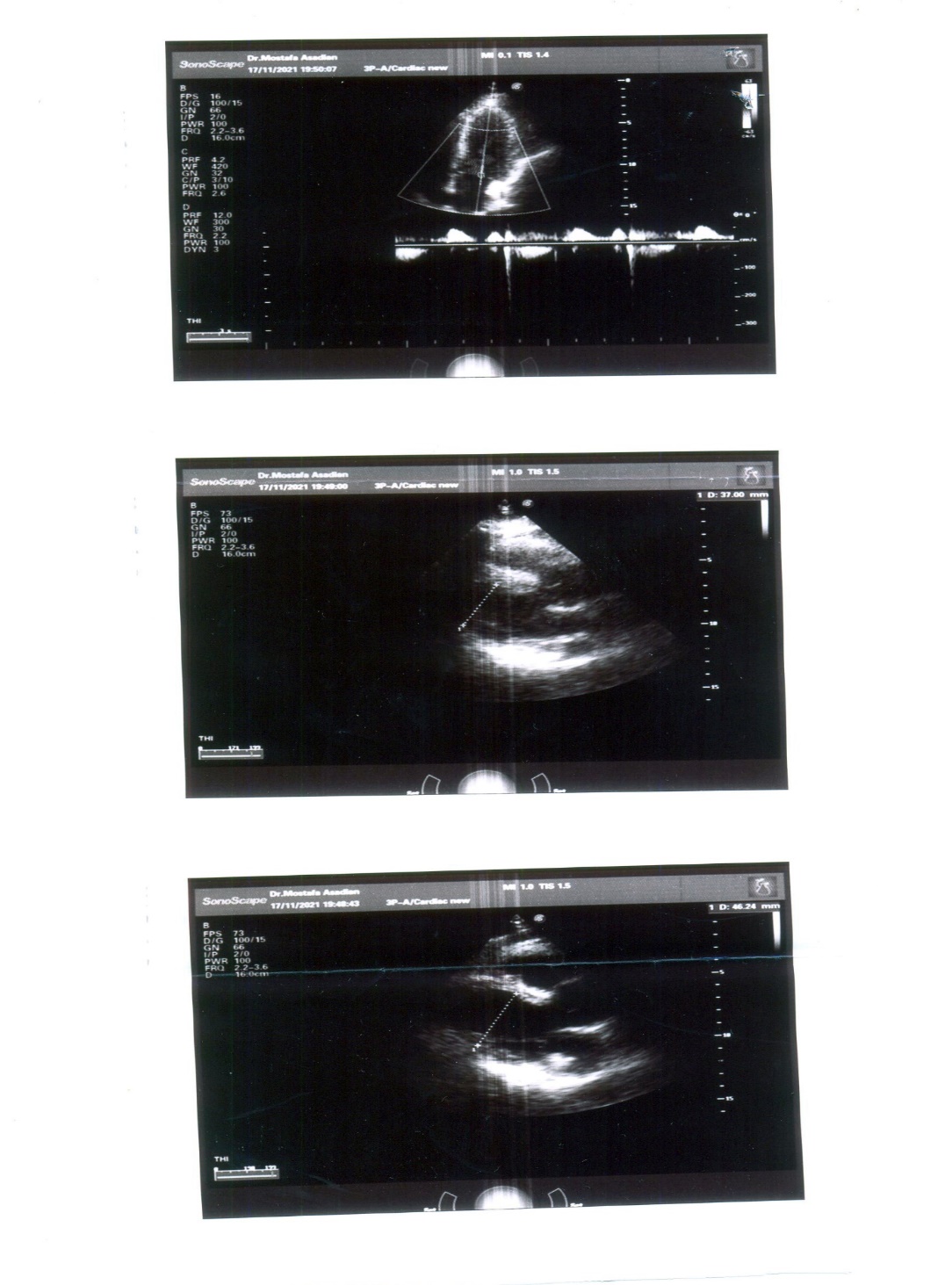
**

**Figure S5** (Echocardiography Pre-test for intervention patient NO.2)

**
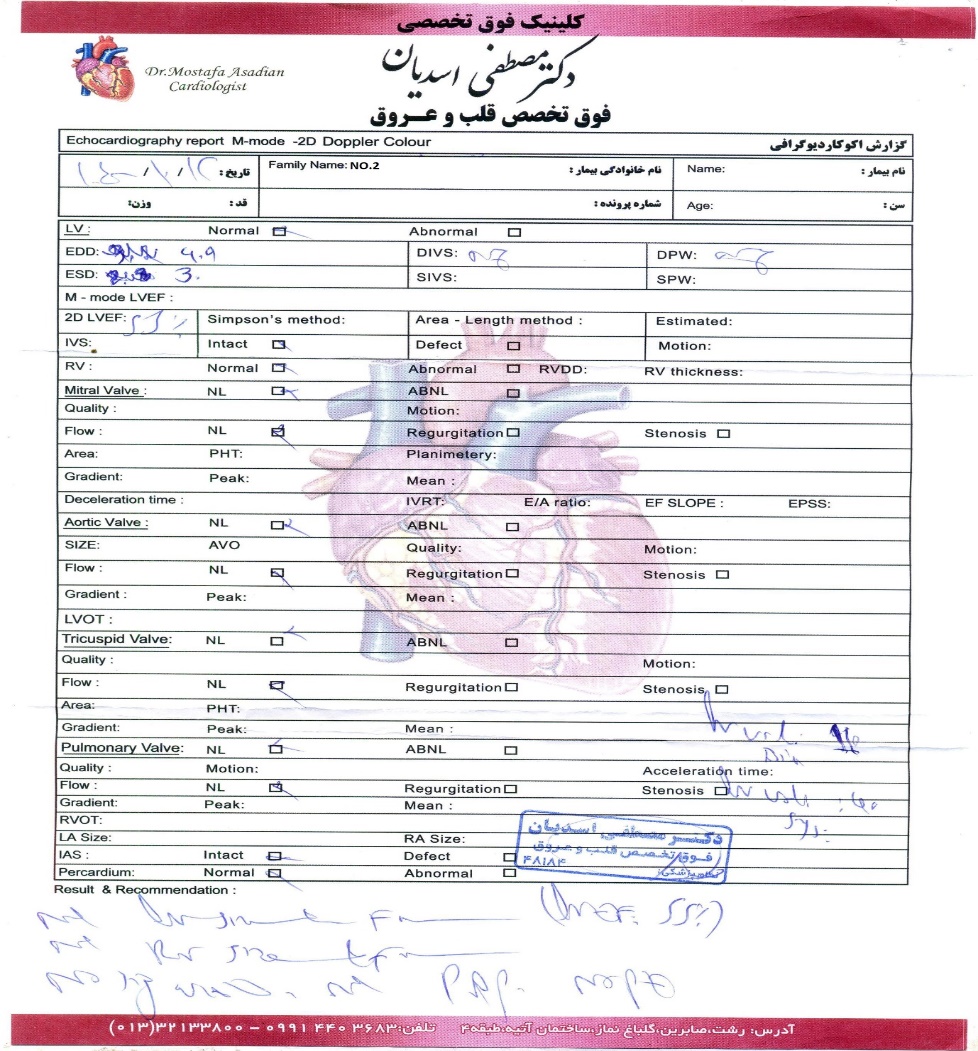
**

**Figure S6** (Echocardiography Post-test for intervention patient NO.2)

**
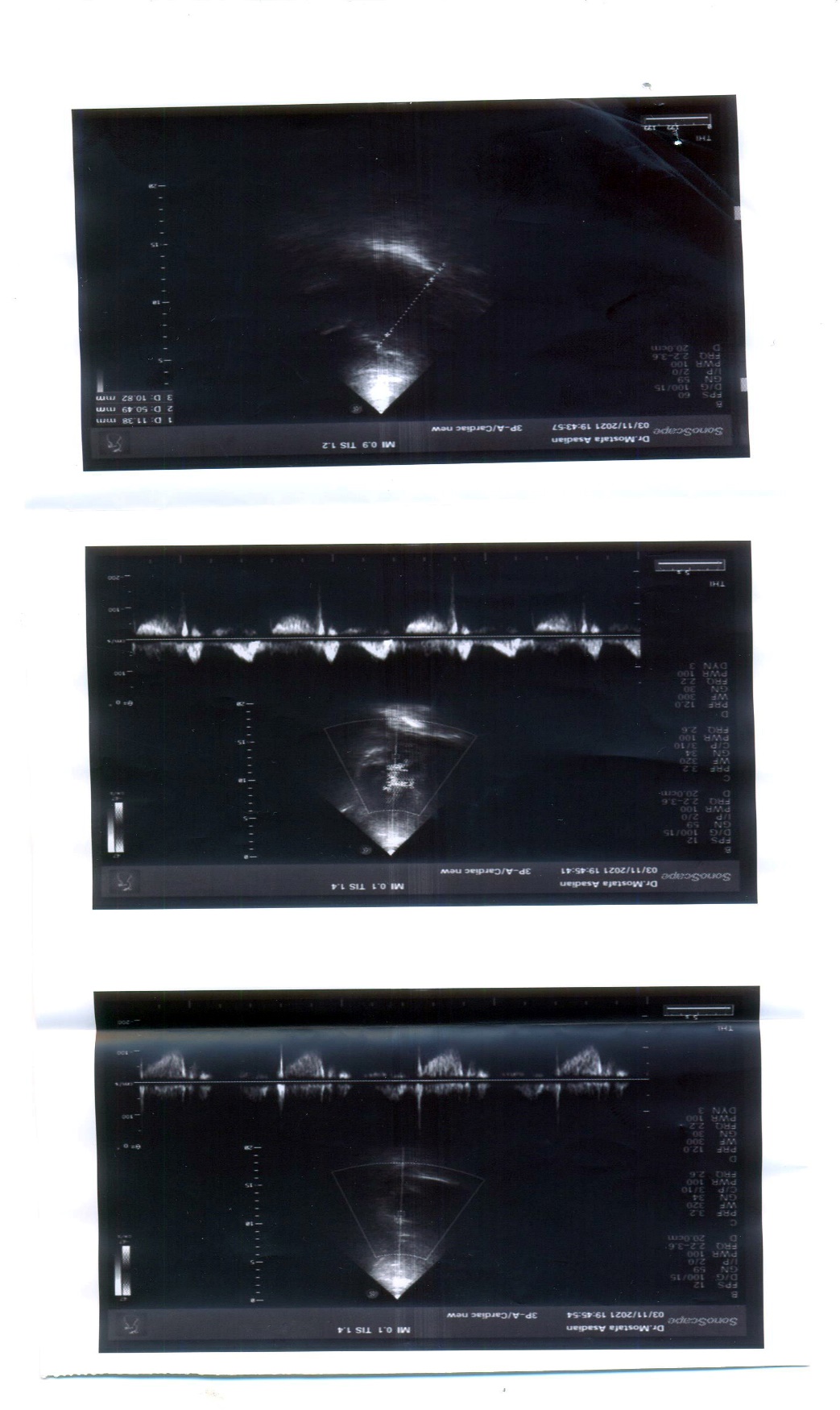
**

**Figure S7** (Echocardiography Pre-test for control patient NO.3)

**
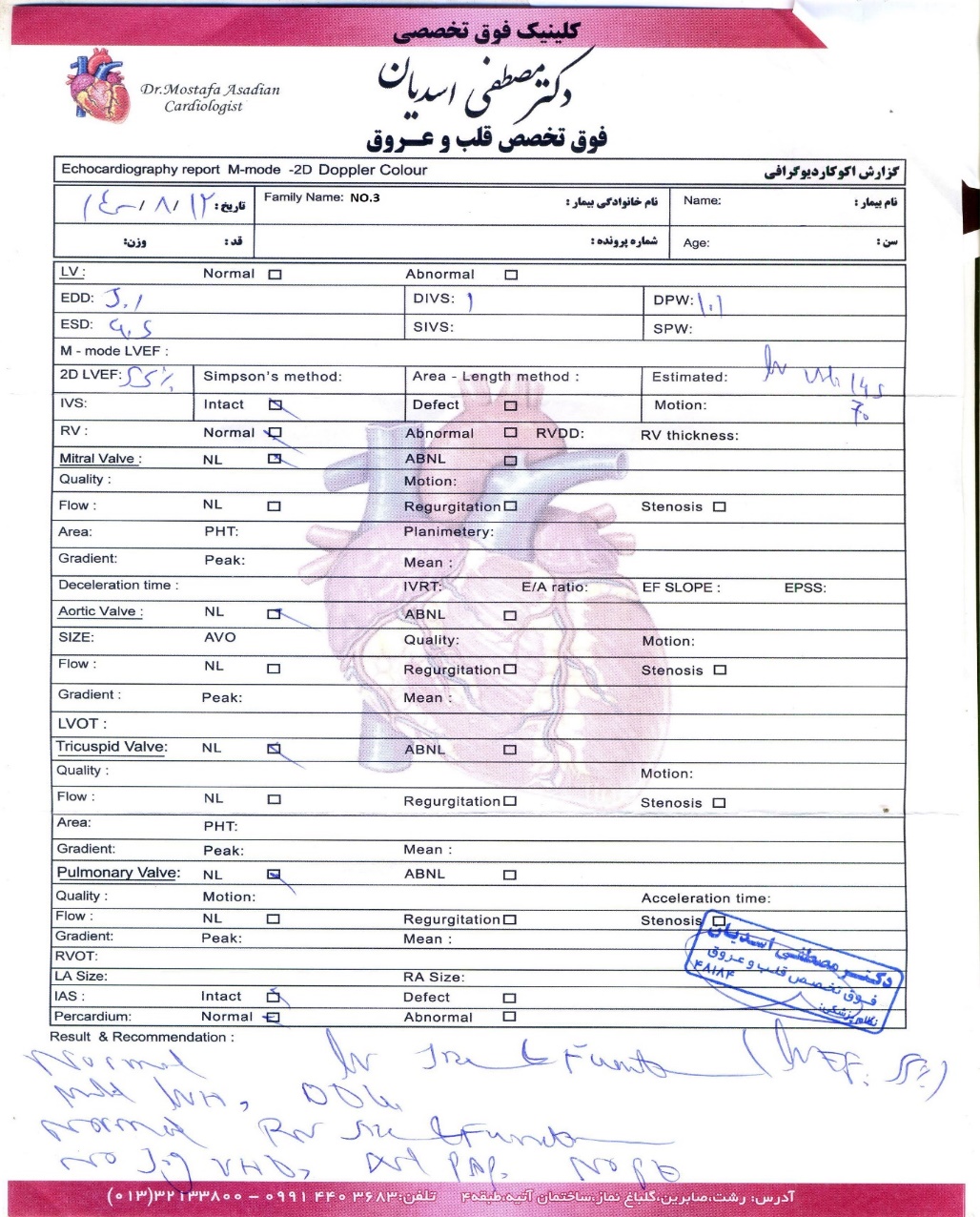
**

**Figure S8** (Echocardiography Pre-test for control patient NO.3)

**
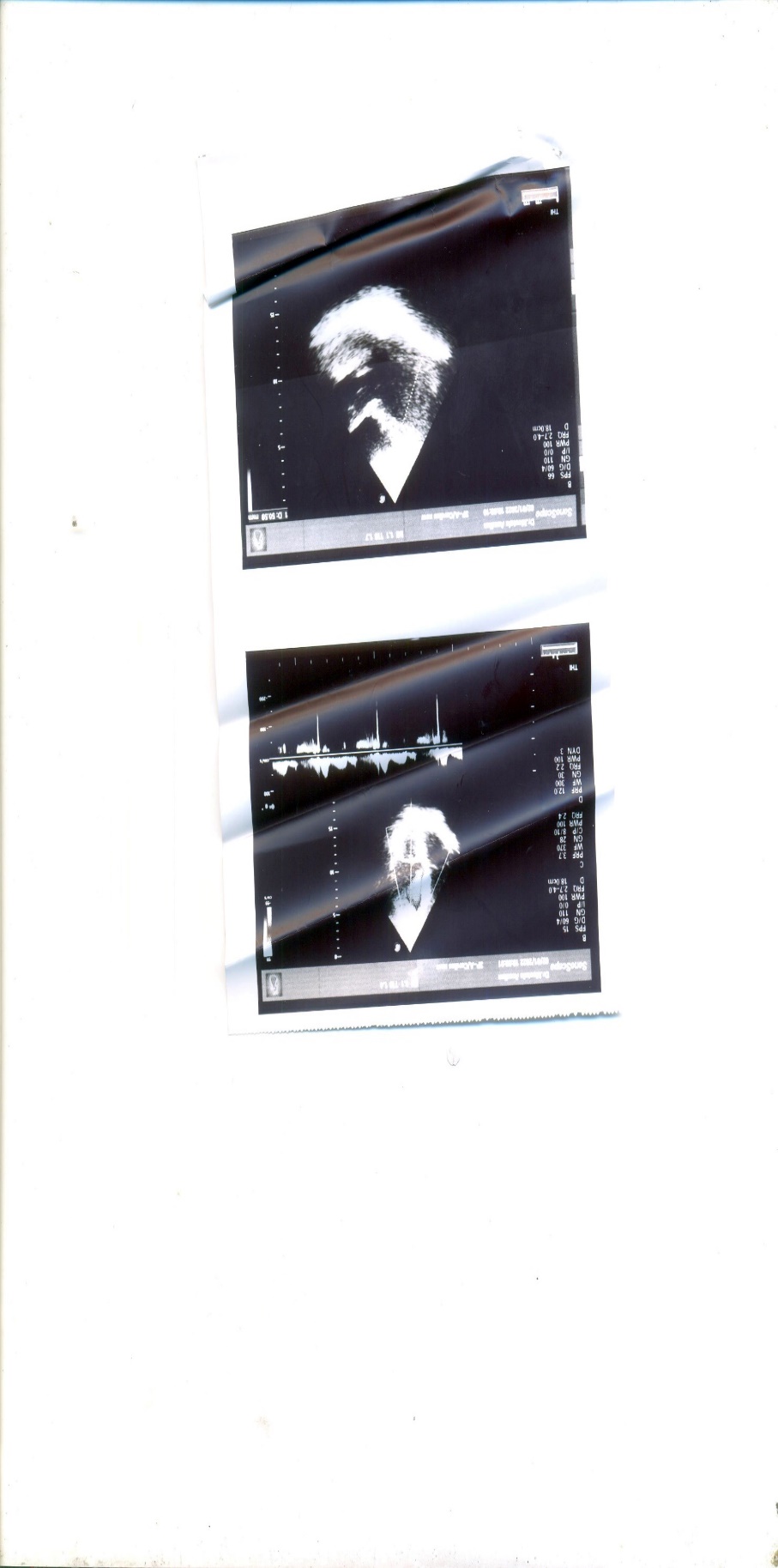
**

**Figure S9** (Echocardiography Post-test for control patient NO.3)

**
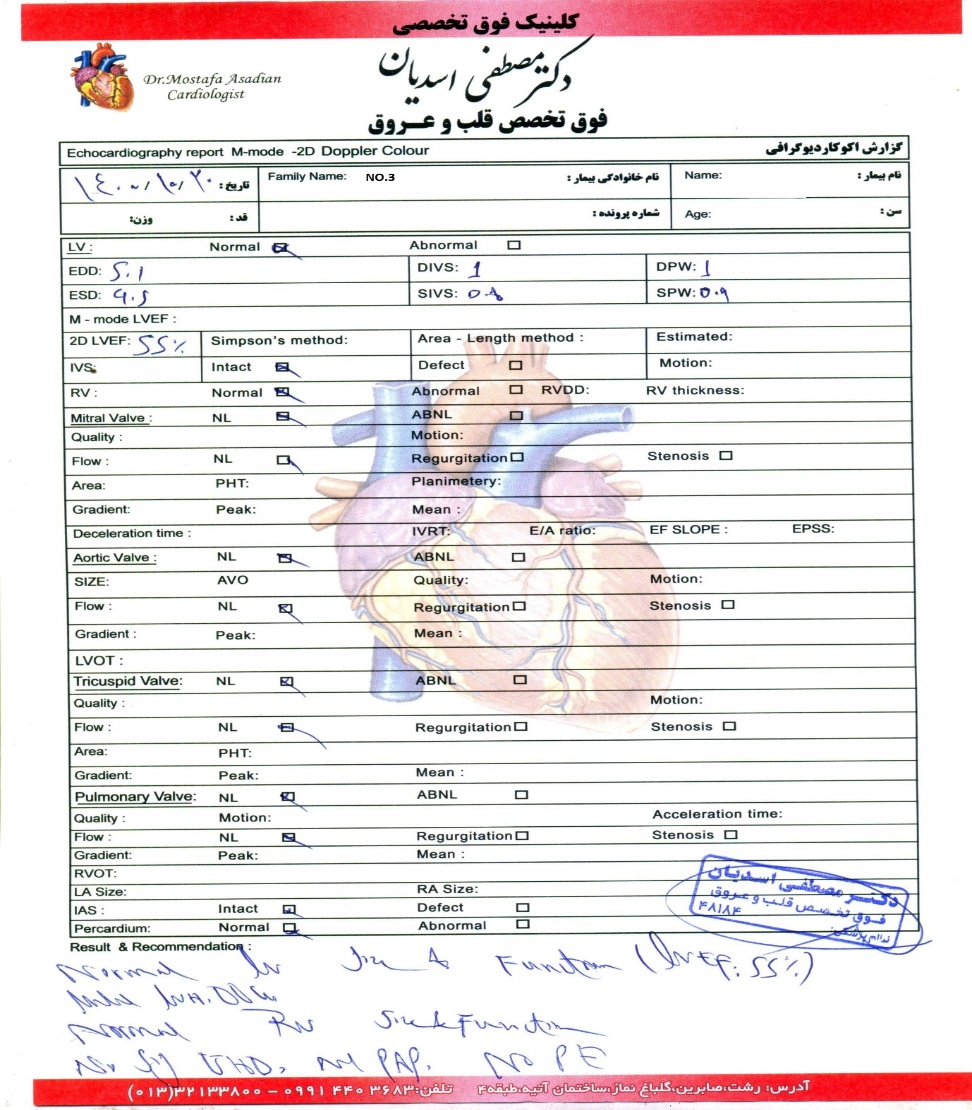
**

**Figure S10** (Echocardiography Post-test for control patient NO.3)

**
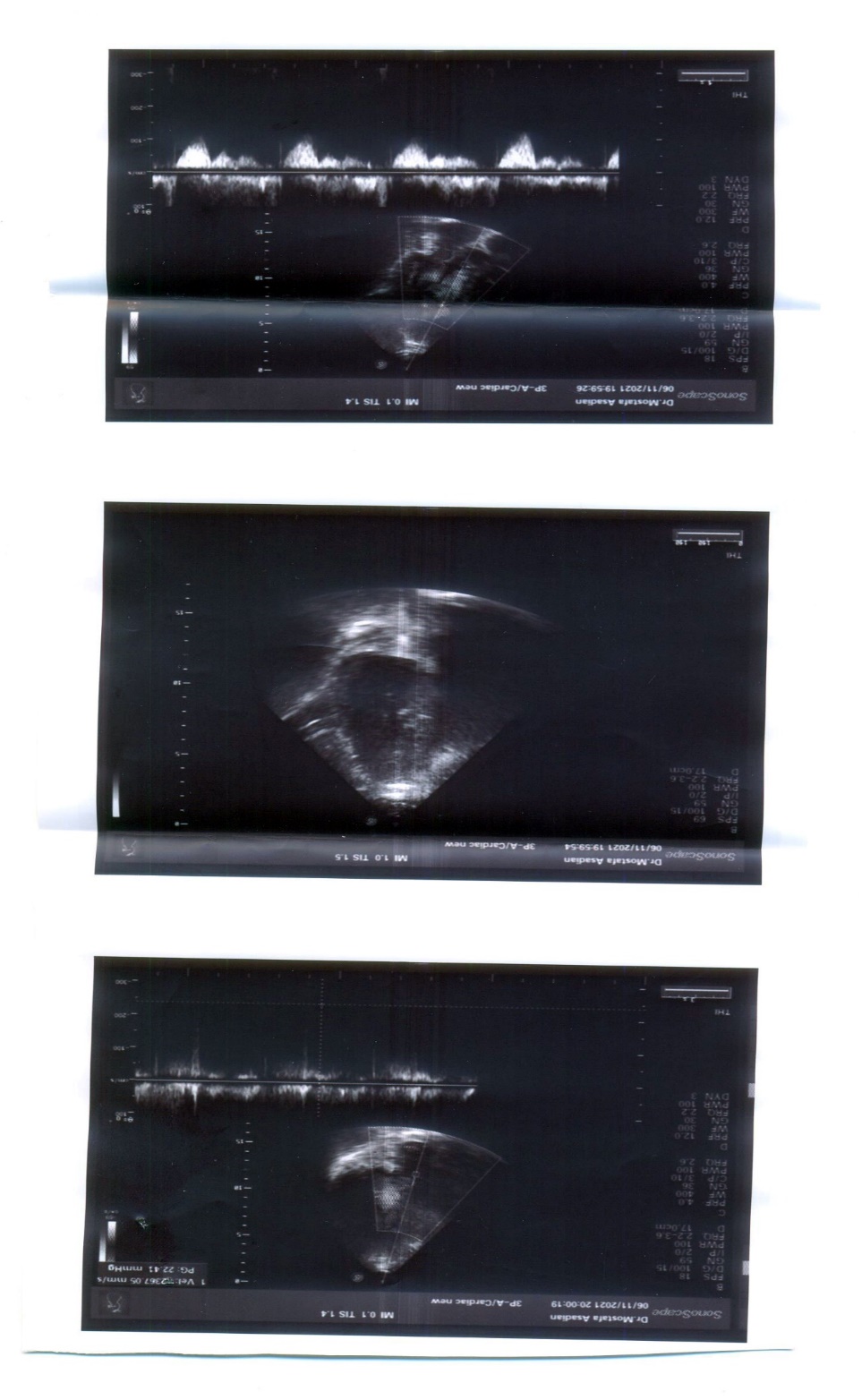
**

**Figure S11** (Echocardiography Pre-test for control patient NO.4)

**
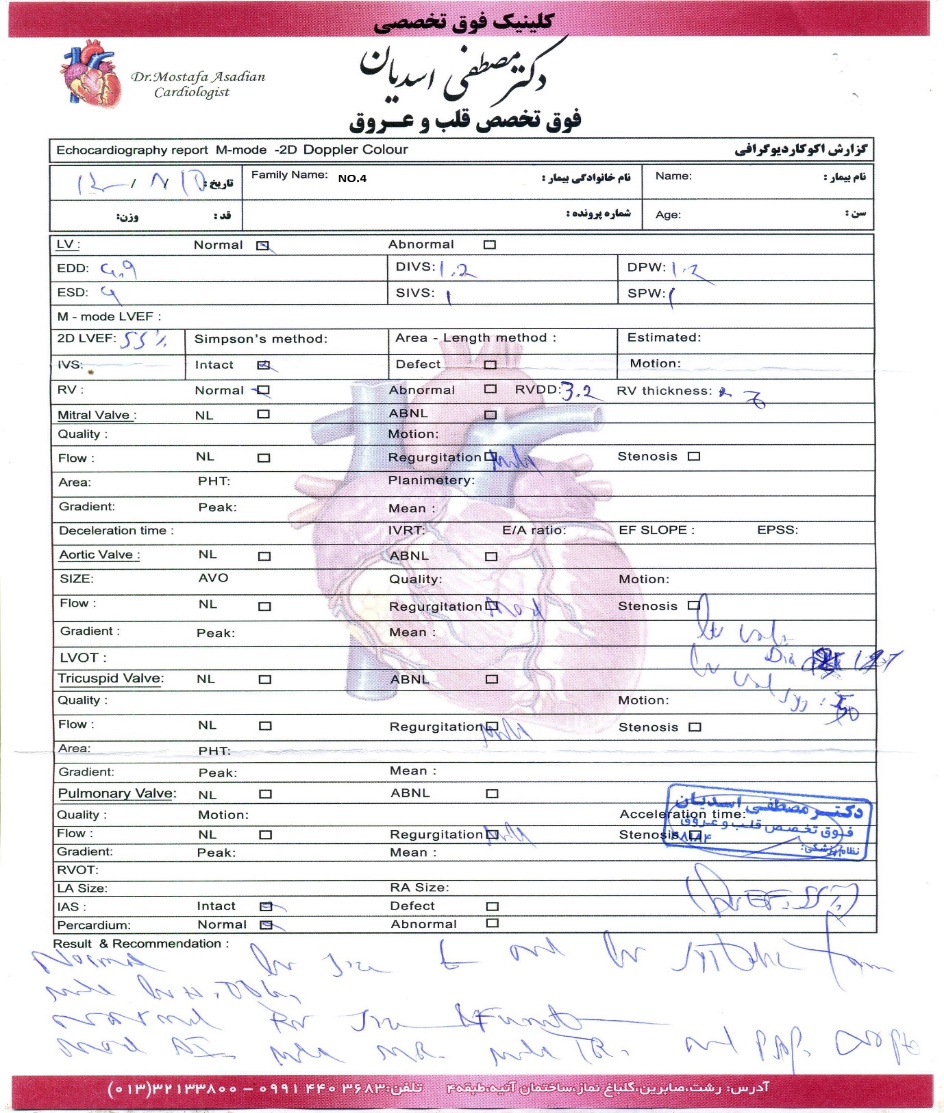
**

**Figure S12** (Echocardiography Pre-test for control patient NO.4)

**
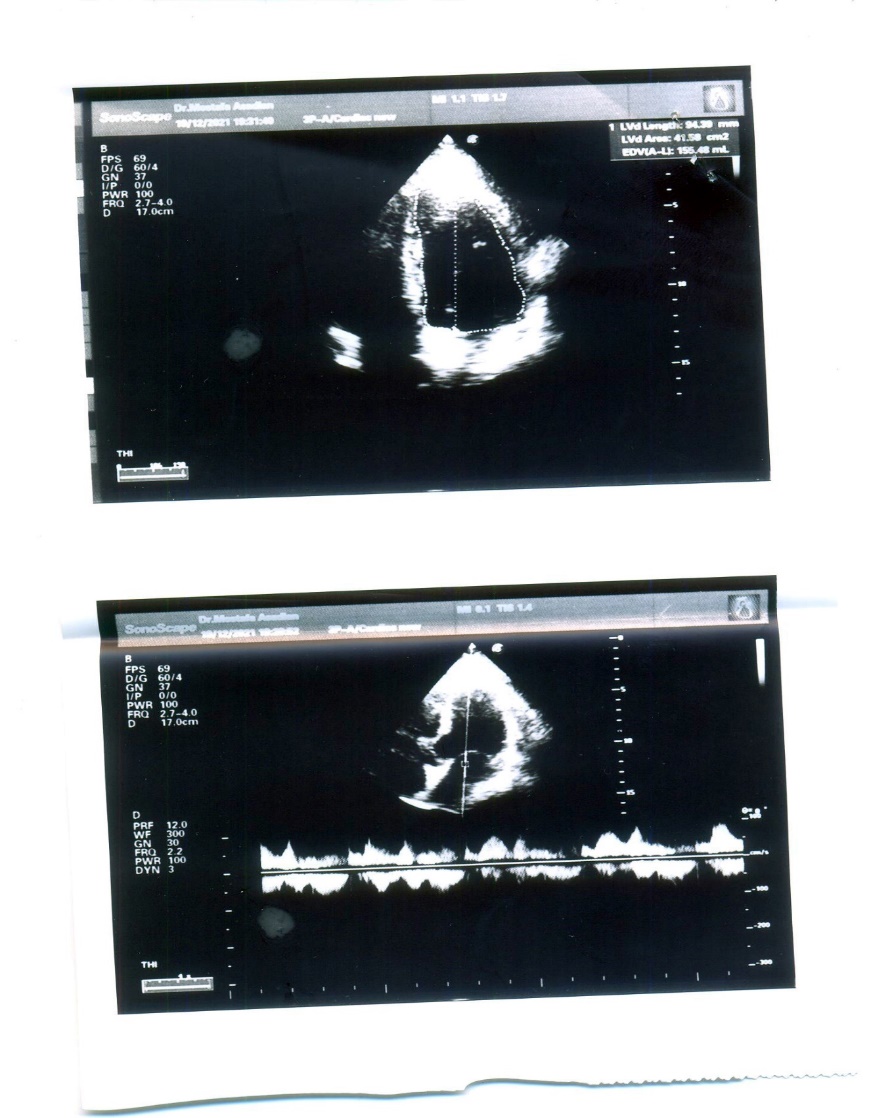
**

**Figure S13** (Echocardiography Post-test for control patient NO.4)

**
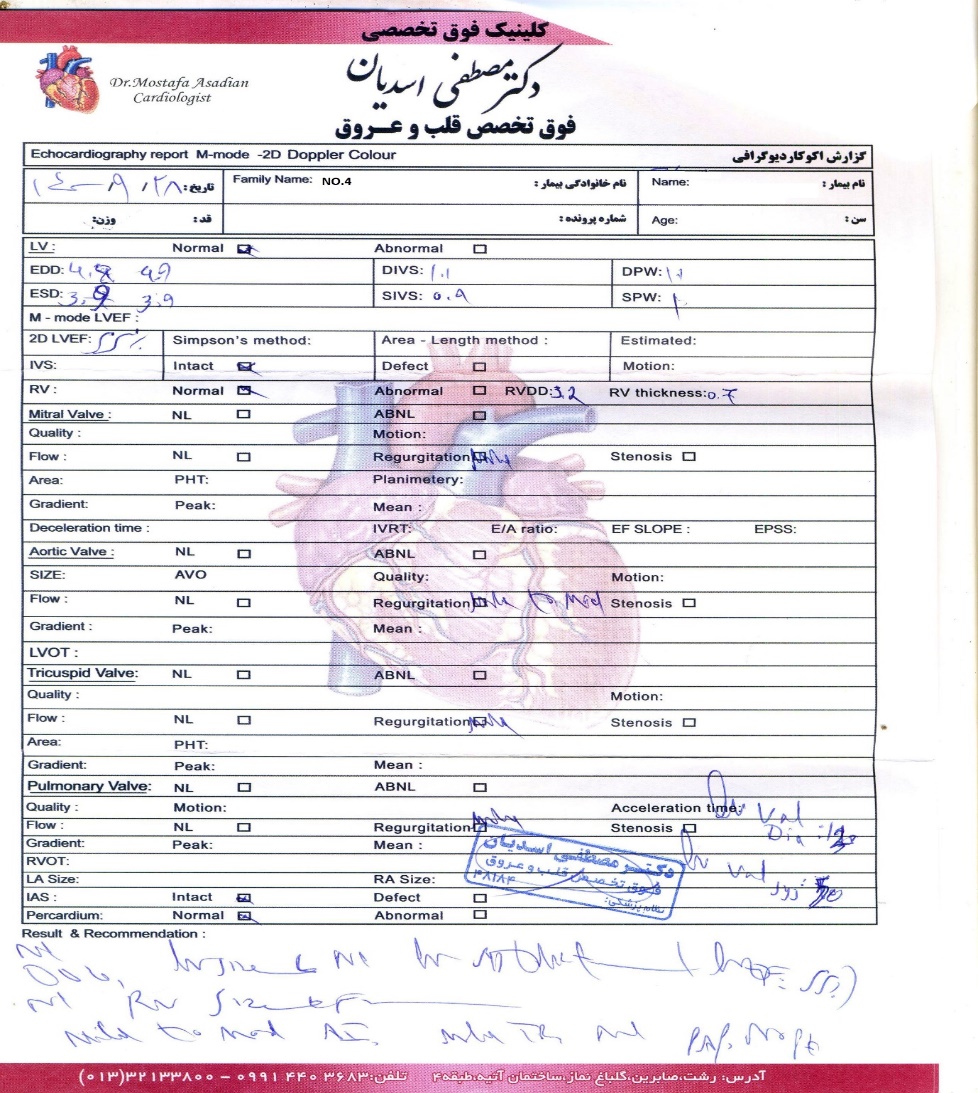
**

**Figure S14** (Echocardiography Pre-test for control patient NO.4)

**
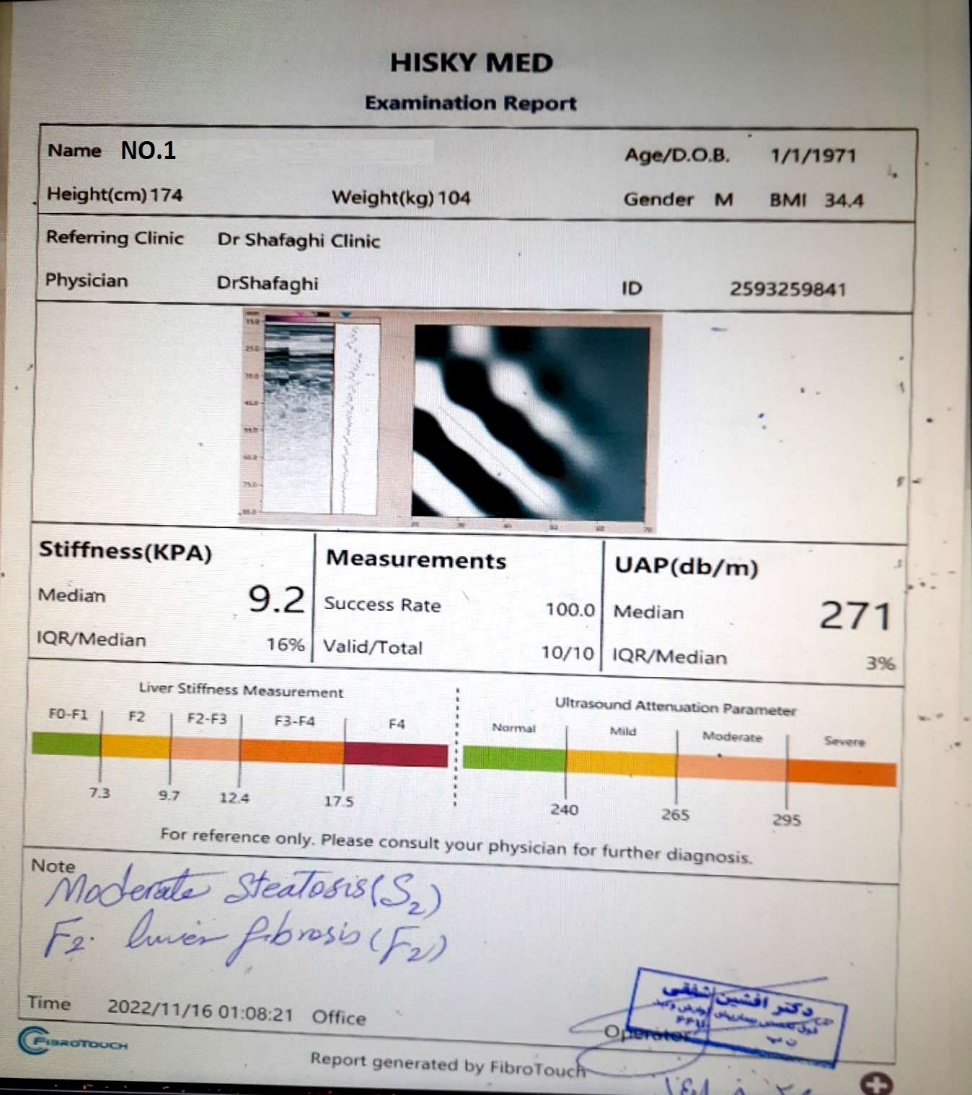
**

**Figure S15** (Fibro Scan Pre-test for intervention patient NO. 1)

**
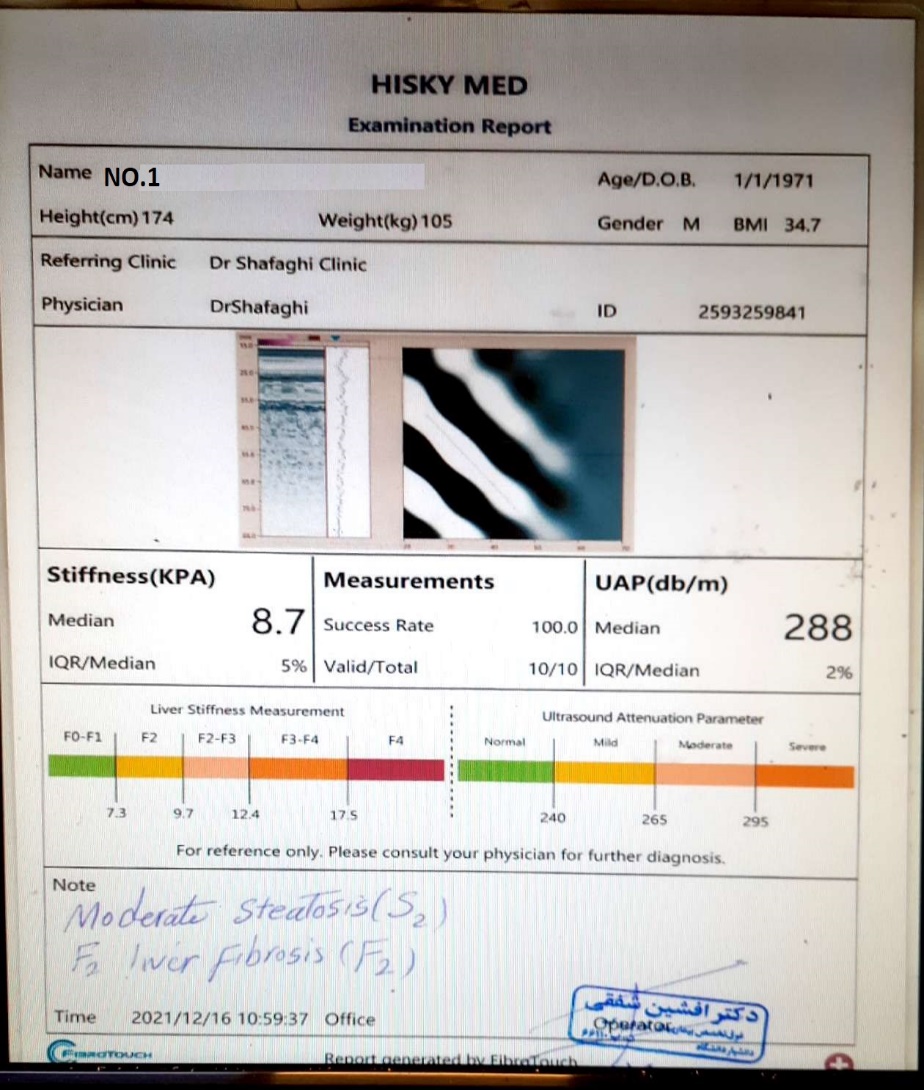
**

**Figure S16** (Fibro Scan post-test for intervention patient NO. 1)

**
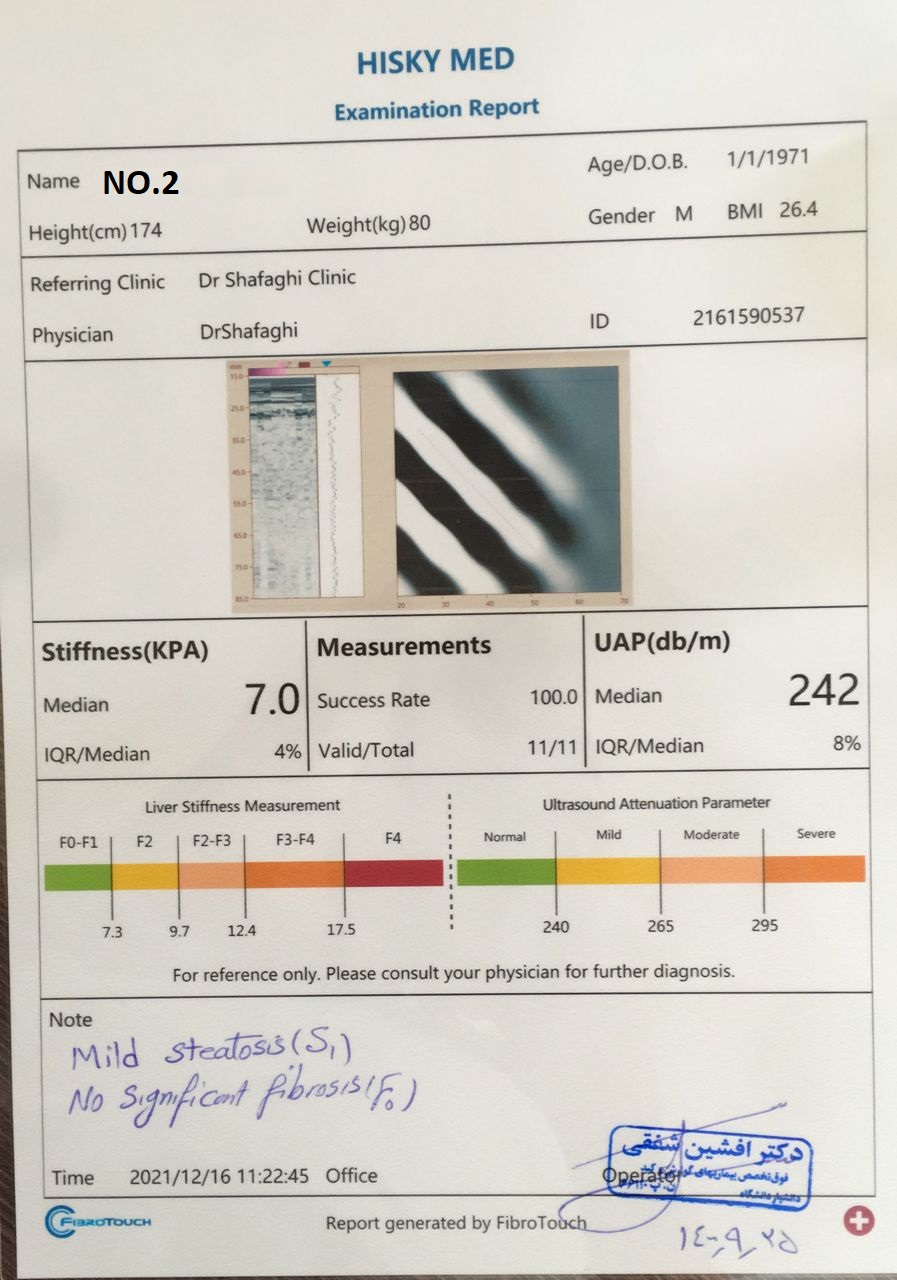
**

**Figure S17** (FibroScan Pre-test for intervention patient NO.2)

**
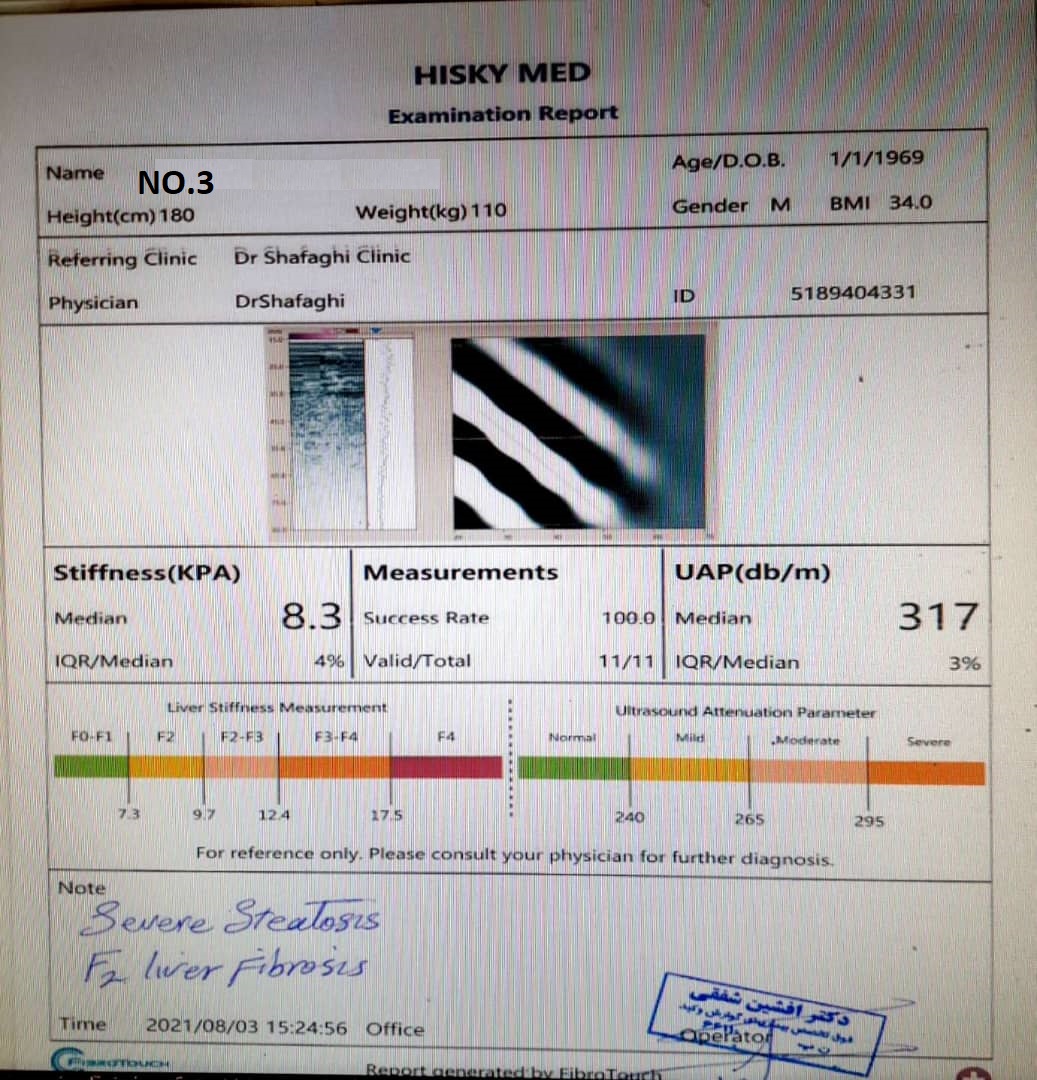
**

**Figure S18** (FibroScan Pre-test for control patient NO. 3)

**
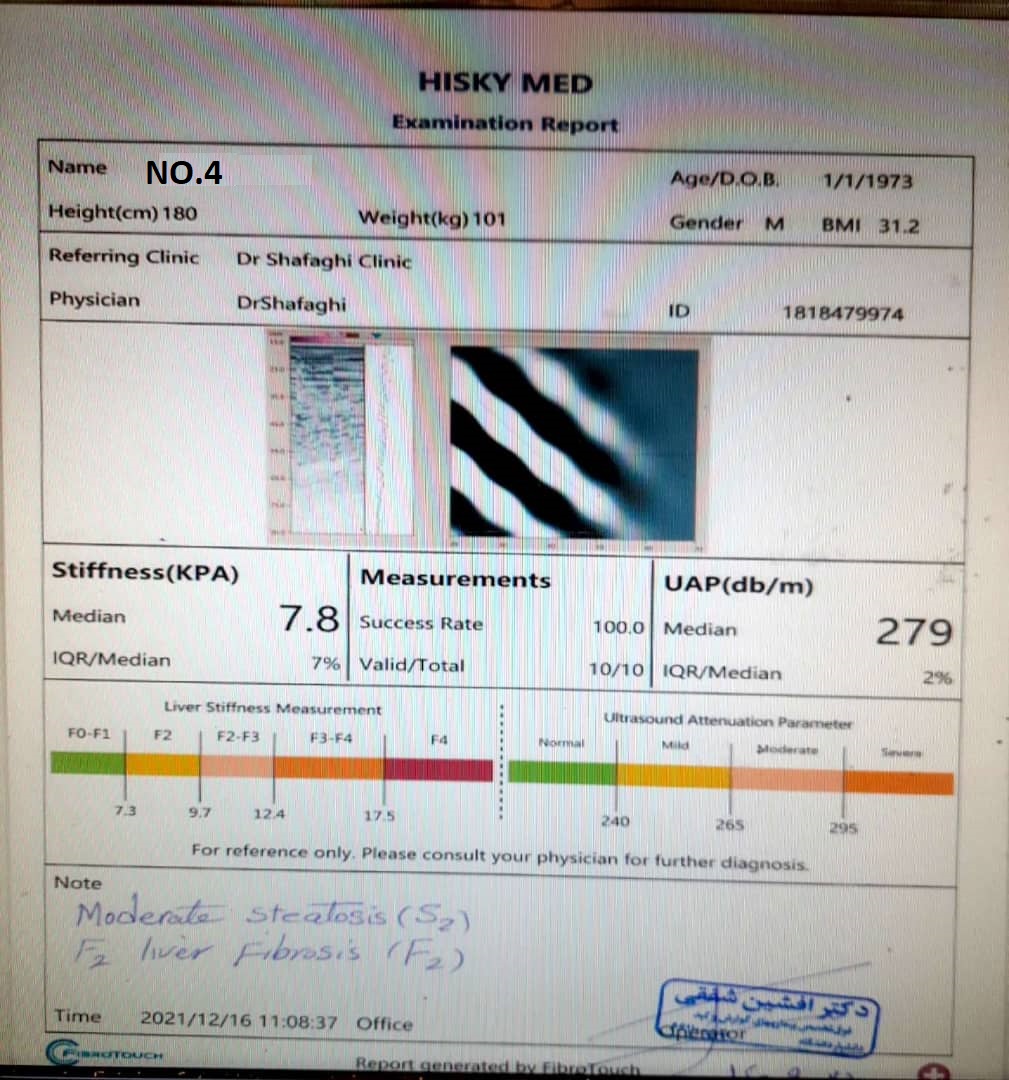
**

**Figure S 19** (FibroScan Pre-test for control patient NO. 4)

**
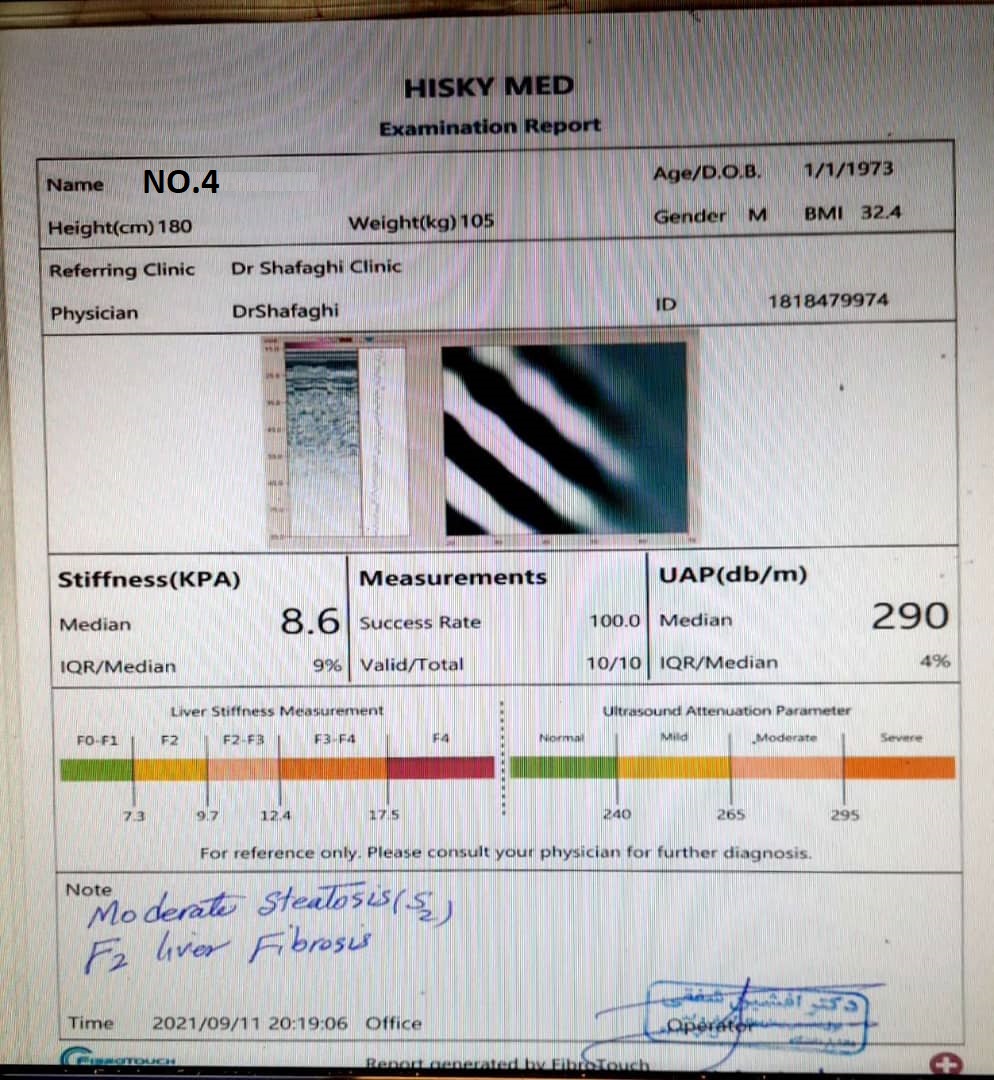
**

**Figure S 20** (FibroScan Post-test for control patient NO. 4)

**
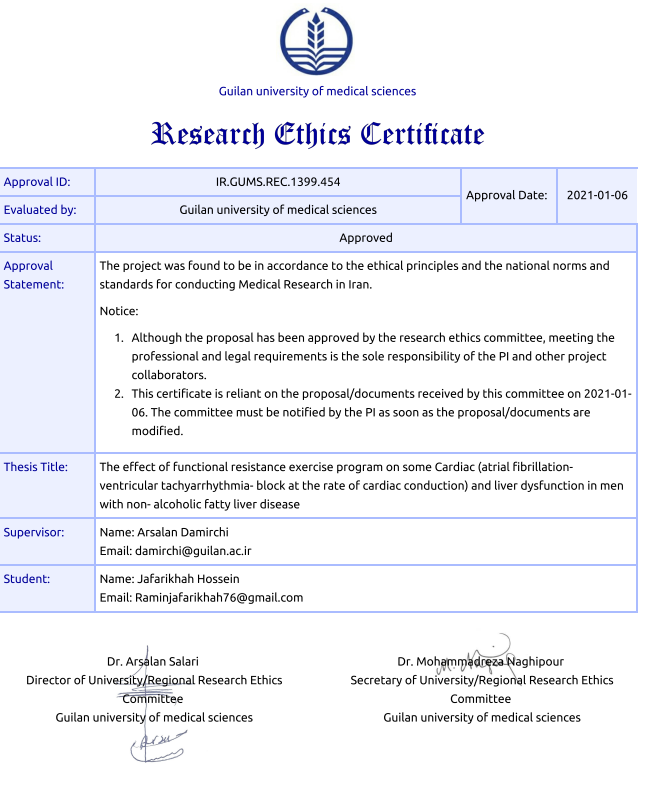
**

**Refrences:**

[S1] Belyavskiy E, Morris DA, Url‐Michitsch M, Verheyen N, Meinitzer A, Radhakrishnan AK, et al. Diastolic stress test echocardiography in patients with suspected heart failure with preserved ejection fraction: a pilot study. 2019;6(1):146-53.

[S2] Tousi SMTR, Faghihi M, Nobakht M, Molazem M, Kalantari E, Azar AD, et al. Improvement of heart failure by human amniotic mesenchymal stromal cell transplantation in rats. 2016;11(3):123.

[S3] Kim D, Konyn P, Cholankeril G, Ahmed AJCG, Hepatology. Physical activity is associated with nonalcoholic fatty liver disease and significant fibrosis measured by fibroscan. 2022;20(6):e1438-e55.
